# Engineered extracellular vesicles reprogram T cells by targeting PD-1 and PHB1 signaling in inflammatory bowel disease

**DOI:** 10.1038/s41392-025-02516-0

**Published:** 2025-12-25

**Authors:** Mi-Kyung Oh, Hyun Sung Park, Dong-Hoon Chae, Aaron Yu, Jae Han Park, Jiyoung Heo, Keonwoo Cho, Jiho Kim, Byeonghwi Lim, Jun-Mo Kim, Jordan E. Axelrad, Kyung Ku Jang, Jong Pil Im, Seong-Joon Koh, Byung-Soo Kim, Kyung-Rok Yu

**Affiliations:** 1 Department of Agricultural Biotechnology and Research Institute of Agriculture and Life Sciences, Seoul National University, Seoul, Korea; 2 Translational Stem Cell Biology Branch, National Heart, Lung, and Blood Institute, National Institutes of Health (NIH), Bethesda, MD USA; 3 Functional Genomics & Bioinformatics Laboratory, Department of Animal Science and Technology, Chung-Ang University, Anseong, Gyeonggi-do Republic of Korea; 4 Division of Gastroenterology and Hepatology, Department of Medicine, NYU Grossman School of Medicine, New York, NY USA; 5 Department of Anatomy, Graduate School of Medical Science, Brain Korea 21 Project, Yonsei University College of Medicine, Seoul, Republic of Korea; 6 Department of Internal Medicine and Liver Research Institute, Seoul National University College of Medicine, Seoul, Korea; 7 Department of Chemical and Biological Engineering, Seoul National University, Seoul, Republic of Korea; 8 Interdisciplinary Program for Bioengineering, Seoul National University, Seoul, Republic of Korea; 9 Institute of Engineering Research, Institute of Chemical Processes, and BioMAX, Seoul National University, Seoul, Republic of Korea; 10 Bio-MAX Institute, Seoul National University, Seoul, Korea

**Keywords:** Mesenchymal stem cells, Immunotherapy

## Abstract

Current therapies for inflammatory bowel disease (IBD) often fail to achieve complete remission and are associated with systemic toxicity owing to their broad immunosuppressive effects. To overcome these limitations, we developed a bioengineered extracellular vesicle (EV) platform that modulates key immune signaling pathways to efficiently restore the T-cell balance in inflamed intestinal tissues. EVs derived from Wharton’s jelly mesenchymal stem cells were engineered to display PD-L1 on their surface and encapsulate miR-27a-3p. Surface PD-L1 engages the PD-1 checkpoint in activated T cells, attenuating T-cell receptor signaling via SHP2-mediated dephosphorylation of ZAP70 and AKT. In parallel, miR-27a-3p suppresses prohibitin 1 (PHB1), a mitochondrial regulator of Th17 cell bioenergetics and inflammatory function, thereby reducing Th17 polarization and increasing the number of FOXP3⁺ regulatory T cells. These dual-targeting EVs preferentially localized to inflamed intestinal tissues via chemokine (CCR2/CXCR4) and PD-1-dependent mechanisms. In humanized mouse models of colitis, these EVs attenuated mucosal inflammation, suppressed effector T-cell responses, and preserved epithelial integrity. In IBD patient-derived colonoid cultures, PD-L1/miR-27a-3p EVs maintained epithelial viability and barrier integrity without inducing cytotoxicity or structural disruption. Transcriptomic and single-cell analyses revealed the downregulation of inflammatory and exhaustion signatures, along with the enrichment of regulatory subsets. Collectively, this study presents a cell-free immunotherapeutic approach that reprograms T cells in inflamed tissues through the PD-1 and mitochondrial signaling pathways while maintaining intestinal epithelial integrity, offering a promising therapeutic strategy for IBD and other T cell-driven inflammatory disorders.

## Introduction

Inflammatory bowel disease (IBD), including Crohn’s disease (CD) and ulcerative colitis (UC), is a chronic relapsing disease characterized by persistent intestinal inflammation and immune dysregulation. Despite the advent of biologic and small molecule agents that act on targets such as TNF-α, IL-12/23, and integrins for treatment, approximately 30–50% of patients exhibit primary nonresponse or develop secondary loss of efficacy.^1,2^ These limitations highlight the need for precision strategies that can restore immune balance without compromising systemic immune surveillance. Although CD and UC differ immunologically, with CD characterized by Th1- and Th17-dominant inflammation through the IL12 and IL23 axis^3,4^ and UC showing type 2 and context- dependent Th17 reactivation,^4,5^ central dysregulation of CD4⁺ T cells is common to both conditions. In both diseases, excessive TNFα, IFNγ, and IL17A disrupt epithelial tight junction integrity and sustain inflammation, while FOXP3⁺ regulatory T-cell insufficiency limits counter regulation.^3^ These findings highlight the importance of reprogramming T-cell function rather than transiently suppressing inflammation.

The PD1/PD-L1 immune checkpoint axis plays a crucial role in peripheral tolerance by attenuating T-cell receptor signaling through SHP2-mediated dephosphorylation of CD3ζ, ZAP70, and CD28, leading to reduced PI3K, AKT, and ERK activation and cytokine release.^6^ Clinical blockade of this axis can trigger colitis, which resembles IBD, emphasizing its role in intestinal immune homeostasis.^7^ In the gut, PDL1 expressed by epithelial and myeloid cells contributes to mucosal tolerance, and altered expression correlates with disease activity. Mucosal PD1 and PDL1 levels are increased in UC and vary by compartment in CD, suggesting that restoring local checkpoint restraint may rebalance mucosal immunity. Engineering extracellular vesicles (EVs) expressing PDL1 is a rational approach for regulating pathogenic T-cell activation within the intestinal microenvironment. In parallel, prohibitin 1 (PHB1), a mitochondrial scaffold protein, regulates intestinal inflammation and T-cell metabolism. PHB1 maintains mitochondrial integrity and barrier function in the intestinal epithelium and Paneth cells,^8^ and supports Th17 bioenergetic programs in CD4⁺ T cells.^9^ MicroRNAs (miRNAs) modulate such immunometabolic pathways—members of the miR-17 ~ 92 family regulate PI3K, AKT, and mTOR signaling and control Th17 and Treg balance,^10^ whereas the miR27 family influences mitochondrial quality control and PHB1 expression.^11,12^ Recent clinical progress with obefazimod, a miR-124 inducer, further supports the therapeutic potential of miRNA-based immune modulation in IBD.^13^ However, the role of miR-27a-3p in human CD4⁺ T cells and its ability to reprogram effector phenotypes remain unclear. In this study, we investigated whether miR-27a-3p modulates T-cell fate via mitochondrial regulators such as PHB1, with implications for rebalancing Th17/Treg dynamics in chronic inflammation.

Mesenchymal stem cell (MSC) therapy has shown promise in IBD, particularly in refractory or fistulizing CD, largely through paracrine mechanisms involving secreted factors and EVs.^14–16^ MSC-derived EVs retain many immunoregulatory properties of their parent cells while offering greater safety, stability, and scalability for clinical use.^17^ Among MSCs from different sources, Wharton’s jelly-derived (WJ) MSCs exhibit potent immunomodulatory activity^18,19^ and express chemokine receptors, such as CCR2 and CXCR4, that facilitate mucosal targeting.^20–22^ Unlike synthetic nanoparticles, MSC EVs serve not only as natural carriers, but also as biologically active components capable of engaging checkpoint receptors and delivering gene-regulatory RNAs into immune cells.

Based on this rationale, we hypothesized that the synchronized modulation of checkpoint and mitochondrial signaling pathways could durably reprogram inflammatory T-cell responses and restore mucosal immune tolerance in patients with IBD. To test this, we developed a dual-functional EV platform that was bioengineered to co-deliver PD-L1 protein and miR-27a-3p. PD-L1 targets the PD-1 checkpoint to suppress surface receptor signaling, whereas miR-27a-3p suppresses PHB1 expression, potentially disrupting mitochondrial function that supports Th17 cells. Using in vitro assays, humanized colitis models, and transcriptomic analyses including single-cell RNA sequencing, we demonstrated that these engineered EVs reprogram inflammatory CD4⁺ T cells, suppress effector polarization, and enhance FOXP3⁺ Treg subsets.

## Results

### BMI1/hypoxia-primed MSC-derived EVs exhibit enhanced immunoregulatory features and alleviate colitis symptoms in vivo

To potentiate the immunoregulatory function of MSC-derived EVs, we engineered WJ-MSCs by combining BMI1 overexpression with hypoxic preconditioning.^23,24^ BMI1 overexpression was successfully confirmed using RT-PCR and western blotting, and was accompanied by decreased expression of the senescence marker p16^INK4a^ and increased proliferative capability of WJ-MSCs (Supplementary Fig. 1a–c). Hypoxic-induced stabilization of HIF-1α further validated successful priming, without negatively impacting cell viability or proliferation (Supplementary Fig. 1d–f). Functionally, BMI1-Hypoxia (BMI1-H)-primed WJ-MSCs demonstrated significantly enhanced immunosuppressive activity, effectively reducing T-cell proliferation compared with normoxic or single-primed controls (Supplementary Fig. 1g).

**Fig. 1:**
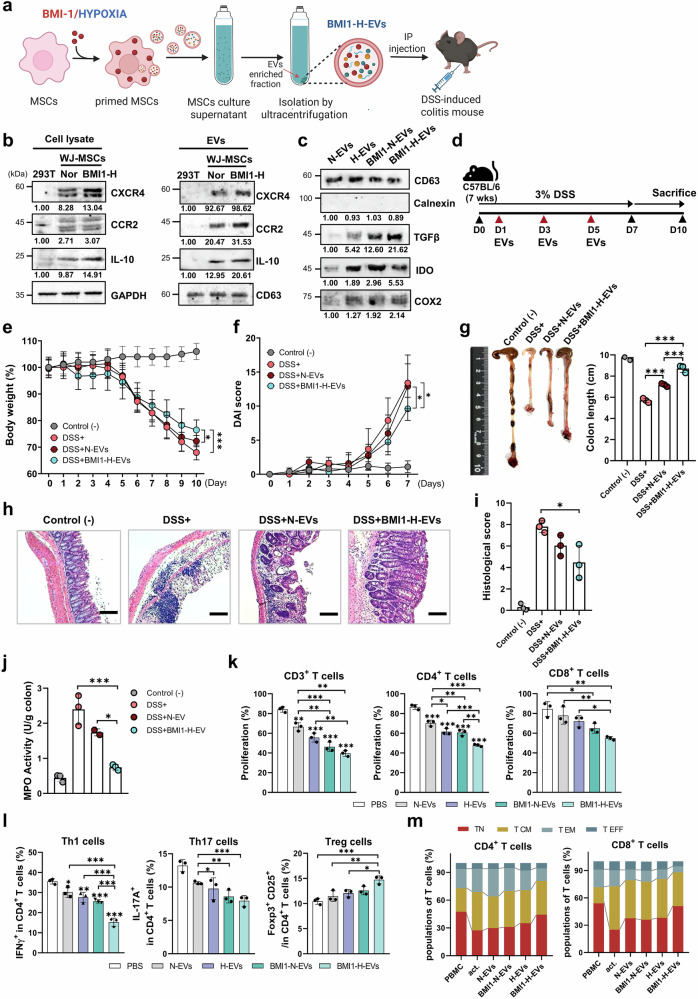
Therapeutic efficacy of BMI/hypoxia-primed Wharton’s jelly-derived MSC (WJ-MSC)-derived extracellular vesicles (EVs) on dextran sulfate sodium (DSS)-induced colitis in mice. **a** Schematic diagram illustrating the generation of EVs from BMI1-overexpressing, hypoxia-conditioned WJ-MSCs (BMI1-H-EVs) and their administration in a (DSS)-induced colitis models (created with BioRender.com). **b** Immunoblot of CXCR4, CCR2, TGF-β, and IL-10 in cell lysates and EVs from 293T, normoxia MSCs, and BMI1-H MSCs. GAPDH and CD63 served as loading control for cell lysate and EVs, respectively. **c** Immunoblot of CD63, TGF-β, IDO, and COX2 in EVs isolated from normoxia-treated (N-EVs), hypoxia-treated (H-EVs), BMI1-overexpressing normoxia-treated (BMI1-N-EVs), and BMI1-overexpressing hypoxia-treated (BMI1-H-EVs) WJ-MSCs. Calnexin was used as a negative marker. Band intensities were quantified using ImageJ and normalized to the corresponding loading controls (cells: GAPDH; EVs: CD63), relative values are shown. **d** Overview of the experimental protocol. Colitis was induced in C57BL/6 mice using 3% DSS for 7 days, and EVs were intraperitoneally injected on days 1, 3, and 5. Mice were sacrificed on day 10 for analysis. **e**, **f** Body weight loss (**e**) and disease activity index (DAI) (**f**) were monitored daily to assess clinical symptoms. **g** Representative colons and quantification of colon length in the indicated group: Control (−), DSS ( + ), DSS + normoxia-treated EVs (N-EVs), and DSS + BMI1-H-EVs. **h**, **i** Hematoxylin and eosin (H&E) staining of colon sections (**h**) and corresponding histological score evaluating tissue architecture, crypt loss, and inflammatory infiltration (**i**). Scale bars: 100 μm. **j** Myeloperoxidase (MPO) activity in murine colon tissue, reflecting neutrophil accumulation. Mouse data are presented as mean ± standard deviation (SD); *n* = 3 mice per group. **k** Carboxyfluorescein succinimidyl ester (CFSE)-based proliferation assay of human PBMC-derived T cells (total, CD4⁺, and CD8⁺) following anti-CD3/CD28 activation (act) and co-culture with naive EVs (N-EVs) or BMI1-H-primed EVs (BMI1-H-EVs). The act. group represents activated PBMCs without EV treatment. **l** Flow cytometric analysis of human CD4^+^ T-cell subsets, including IFN-γ⁺ Th1, IL-17A⁺ Th17, and CD25⁺FOXP3⁺ regulatory T cells (Tregs), following EV treatment. **m** Flow cytometric analysis of memory T-cell phenotypes with human CD4^+^ and CD8^+^ populations based on CD45RA and CCR7expression: naive (TN, CD45RA^+^CCR7^+^), central memory (TCM, CD45RA^−^CCR7^+^), effector memory (TEM, CD45RA^−^CCR7^−^), and terminal effector (TEFF, CD45RA^+^CCR7^−^) subsets. CD63 was included as an EV marker. Human data are shown as mean ± SD; *n* = 3 independent donors (biological replicates), each assayed in technical triplicate. Time-course panels were analyzed using two-way repeated-measures ANOVA (treatment × day) with Sidak’s multiple comparisons at each day and single time-point panels with three or more groups used one-way ANOVA with Sidak’s correction. *p < 0.05, **p < 0.01, ***p < 0.001

Next, we generated EVs from BMI1-H-primed WJ-MSCs (BMI1-H-EVs) and administered them to a dextran sulfate sodium (DSS)-induced mouse model of colitis (Fig. 1a). BMI1-H-EVs displayed a typical cup-shaped morphology, observed using transmission electron microscopy (TEM) and CD81 expression was confirmed by immunogold labeling (Supplementary Fig. 2a). Western blot analysis showed that BMI1-H-EVs retained canonical EV markers (CD9, CD63, and CD81) and exhibited increased expression of immunomodulatory proteins, including TGF-β, IL-10, IDO, and COX2 (Fig. 1b, c and Supplementary Fig. 2b–d). Notably, WJ-MSC-derived EVs expressed markedly higher levels of CCR2 and CXCR4 than 293T-derived EVs in both cellular lysates and EV fractions (Fig. 1b). This elevated expression of homing-associated receptors supports the preferential use of WJ-MSCs over 293 T cells as EV-producing platforms because of their enhanced potential to target inflamed intestinal tissues. Nanoparticle tracking analysis (NTA) confirmed comparable size distributions across the EV groups, with a higher yield observed for BMI1-H-EVs (Supplementary Fig. 2e).

In a dextran sulfate sodium (DSS)-induced colitis mouse model, intraperitoneal administration of BMI1-H-EVs significantly mitigated disease symptoms (Fig. 1d). Mice treated with BMI1-H-EVs displayed significantly attenuated weight loss and reduced disease activity index (DAI) scores compared with the DSS-only or normoxia EVs (N-EV) groups (Fig. 1e, f). Colon shortening was markedly alleviated (Fig. 1g), and histological analysis revealed a preserved epithelial architecture with reduced crypt loss and inflammatory infiltration (Fig. 1h, i). Myeloperoxidase (MPO) activity, which is indicative of neutrophil accumulation, was significantly lower in the BMI1-H-EV group (Fig. 1j). Transcriptional profiling revealed modest overall T cell-related changes in DSS-induced colitis mouse model, consistent with its innate-dominant biology. However, BMI1-H-EV treatment, reduced Tbx21 and Rorc levels and increased Foxp3 and Il10 levels (Supplementary Fig. 3a). In parallel, lamina propria analysis revealed that although total macrophage numbers were unchanged, EVs promoted an M2-like phenotype with higher CD206 and ARG1 expression (Supplementary Fig. 3b–e). In in vitro studies, co-culture of BMI1-H-EVs with activated human PBMCs resulted in robust suppression of CD4⁺ and CD8 + T-cell proliferation (Fig. 1k and Supplementary Fig. 4a, b). Flow cytometric analysis further revealed reduced frequencies of IFN-γ^+^ Th1 and IL-17A^+^ Th17 cells, along with increased CD25^+^ FOXP3^+^ regulatory T cells (Tregs), indicating a shift toward an anti-inflammatory phenotype (Fig. 1l and Supplementary Fig. 4c, d). Notably, EV treatment also skewed memory T-cell composition toward naive phenotypes, while reducing effector memory (TEM) and terminal effector (TEFF) subsets (Fig. 1m and Supplementary Fig. 4e, f), suggesting effective reprogramming of mucosal immune dynamics.

### BMI1-H-primed EVs exert immunomodulatory effects via miR-27a-3p-mediated T cell reprogramming

**Fig. 2:**
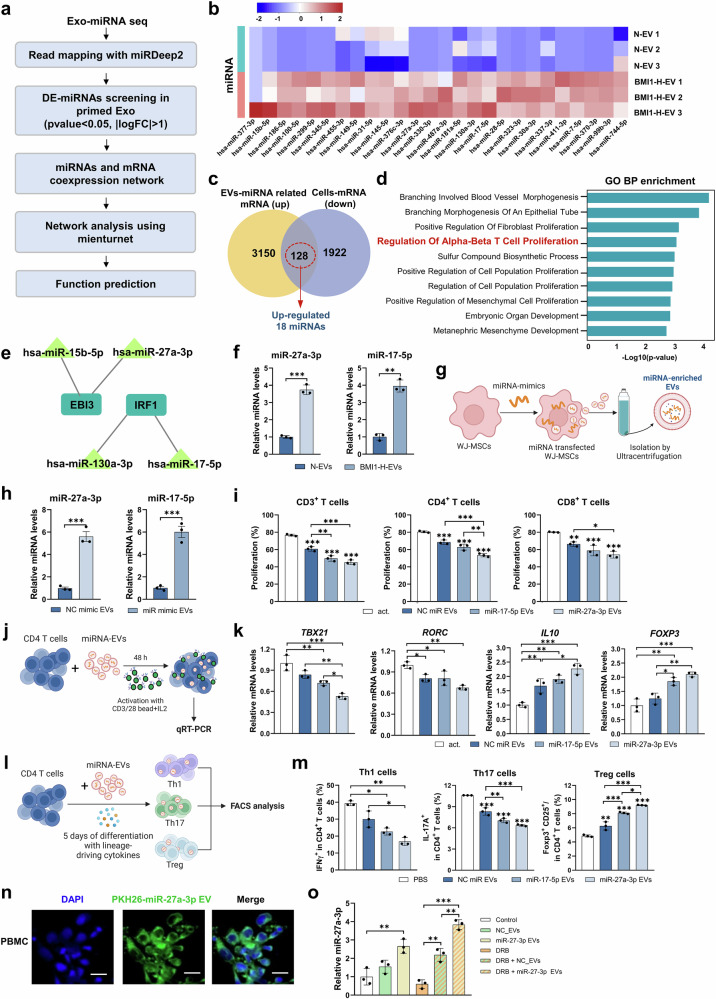
Identification of immunoregulatory miRNAs enriched in engineered WJ-MSC-derived EVs and their immunomodulatory effects on human T cells. **a** Schematic overview of the experimental workflow for miRNA profiling and integrative analysis of BMI1-H-EVs compared with N-EVs. **b** Heatmap of differentially expressed miRNA (DE-miRNAs) between BMI1-H-EVs and N-EVs (fold change ≥2, *p* < 0.05; *n* = 3). **c** Venn diagram showing 128 overlapping genes identified from the intersection of target genes predicted from EV-enriched miRNAs and mRNAs downregulated in BMI1-H-MSCs. **d** Gene Ontology (GO) Biological Process enrichment analysis of target genes associated with 18 DE-miRNAs. **e** miRNA–mRNA interaction network of four hub miRNA (miR-15b-5p, miR-17-5p, miR-27a-3p, and miR-130a-3p) and their predicted targets (EBI3 and IRF1). **f** qRT-PCR validation of miR-17-5p and miR-27a-3p expression levels in BMI1-H-EVs compared with those in N-EVs. **g** Schematic illustration of EV production from WJ-MSCs transfected with synthetic miRNA mimics (miR-17-5p, miR-27a-3p, and negative control (NC)) (created with BioRender.com). **h** qRT-PCR analysis of miR-17-5p and miR-27a-3p expression in EVs derived from mimic-transfected WJ-MSCs. **i** CFSE-based proliferation assay of human PBMC-derived T cells (total, CD4⁺, and CD8⁺) following anti-CD3/CD28 activation (act) and co-culture with miRNA-enriched EVs. The act group represents activated human PBMCs without EV treatment. **j** Schematic of in vitro human CD4⁺ T-cell activation using anti-CD3/28 antibodies and EV treatment (created with BioRender.com). **k** qRT-PCR analysis of *TBX21*, *RORC*, *FOXP3*, and *IL10* expression in human CD4⁺ T cells after treatment with miRNA-enriched EVs. **l** Schematic overview of cytokine-driven polarization assays combined with EV treatment (created with BioRender.com). **m** Flow cyto**m**etric analysis of IFN-γ⁺ Th1, IL-17A⁺ Th17, and CD25⁺FOXP3⁺ Treg population in human CD4⁺ T cells treated with miR-17-5p- or miR-27a-3p-enriched EVs. **n** Representative fluorescence images showing uptake of PKH26-labeled EVs (green) by human PBMCs. Nuclei were counterstained with DAPI (blue). Scale bars: 20 μm. **o** qRT-PCR analysis of intracellular miR-27a-3p in PBMCs pretreated with DRB (RNA polymerase II inhibitor, 20 μM), followed by anti-CD3/CD28 activation and EV treatment. Data represent *n* = 3 independent human PBMC donors (biological replicates), each measured in triplicate technical replicates. Statistical significance was assessed using paired Student’s t-test for two groups or one-way ANOVA with Tukey’s multiple comparisons test for more than two groups. *p < 0.05, **p < 0.01, ***p < 0.001

To investigate the molecular basis of the enhanced immunomodulatory properties conferred by BMI1-H priming, we performed integrative microRNA (miRNA) and transcriptomic profiling. Comparative small RNA-seq analysis revealed 26 miRNAs that were significantly enriched in BMI1-H-EVs compared with N-EVs (Fig. 2a, b). Bulk RNA-seq analysis of BMI1-H-primed WJ-MSCs identified 1922 downregulated transcripts, 128 of which were predicted to be targets of 18 differentially expressed miRNAs (Fig. 2c and Supplementary Fig. 5a). Gene ontology (GO) enrichment analysis indicated that these targets were significantly associated with T-cell activation and proliferation pathways (Fig. 2d). Network modeling focusing on the GO term ‘regulation of alpha-beta T cell proliferation’, identified miR-27a-3p, miR-17-5p, miR-130a-3p, and miR-15b-5p as central regulators, targeting immunomodulatory genes such as *EBI3* and *IRF1* (Fig. 2e and Supplementary Fig. 5b). qRT-PCR confirmed the upregulation of these candidate miRNAs in BMI1-H–primed WJ-MSCs (Supplementary Fig. 5c). To evaluate their functional roles, synthetic miRNA mimics were transfected into WJ-MSCs (Supplementary Fig. 5d, e) and the resulting EVs were assessed for T-cell suppression. Among these candidates, miR-27a-3p and miR-17-5p mimics significantly inhibited the proliferation of activated CD4^+^ and CD8^+^ T cells (Supplementary Fig. 5f).

Subsequent analyses confirmed the selective enrichment of miR-27a-3p and miR-17-5p in BMI1-H-EVs (Fig. 2f). When loaded onto naive EVs, synthetic miR-27a-3p and miR-17-5p recapitulated their suppressive effects on T-cell proliferation in vitro (Fig. 2g–i and Supplementary Fig. 6a). In CD4^+^ T cells stimulated with anti-CD3/CD28, miRNA-enriched EVs led to the downregulation of *TBX21* and *RORC* and upregulation of *FOXP3* and *IL10*, indicating regulatory T-cell polarization (Fig. 2j, k). Furthermore, under Th1- and Th17-polarizing cytokine conditions, miR-27a-3p-enriched EVs robustly suppressed effector differentiation and enhanced Treg induction (Fig. 2l, m, and Supplementary Fig. 6b). Notably, miR-27a-3p consistently outperformed miR-17-5p in suppressive efficacy, in line with previous reports linking miR-27a-3p to immune and intestinal regulation.^25,26^ These findings establish that miR-27a-3p is a central component of the BMI1-H-EV therapeutic effect, mediating effector suppression and regulatory T-cell reprogramming. To confirm the EV-mediated transfer of miR-27a-3p into target cells, PKH26-labeled EVs were visualized in human PBMCs and internalization was confirmed using the RNA polymerase II inhibitor DRB, which abrogated endogenous transcription (Fig. 2n, o). These findings establish that miR-27a-3p is a central component of the therapeutic effect exerted by BMI1-H-EV, mediating effector suppression and regulatory T-cell reprogramming.

### miR-27a-3p-enriched EVs suppress T cell proliferation and differentiation by directly targeting PHB1

**Fig. 3:**
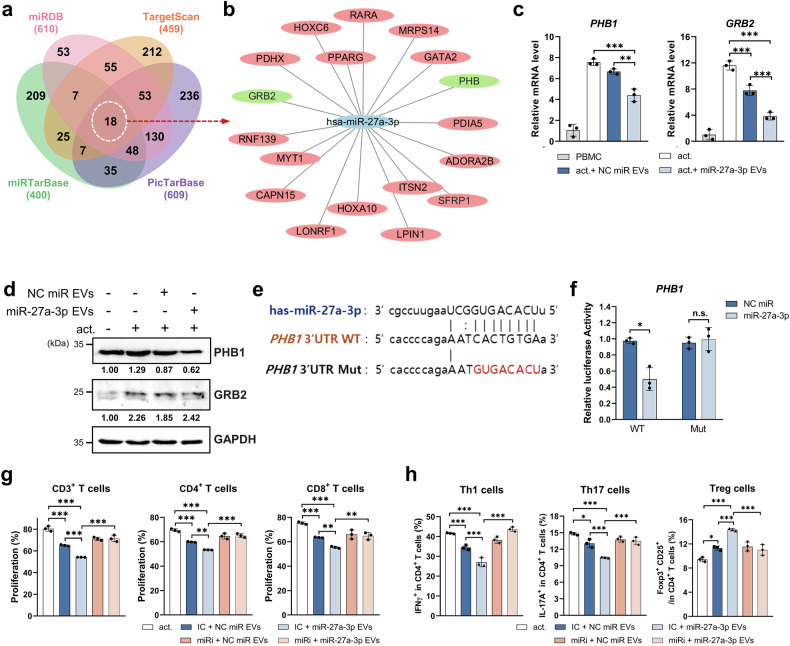
miR-27a-3p-enriched EVs suppress T-cell proliferation and differentiation via targeting PHB1. **a** Venn diagram of predicted miR-27a-3p target genes identified from four databases: TargetScan, miRTarBase, miRDB, and picTarBase. **b** Network visualization of predicted targets of miR-27a-3p. **c** qRT-PCR analysis of *PHB1* and *GRB2* mRNA expression in anti-CD3/CD28-activated human PBMCs treated with control or miR-27a-3p-enriched EVs. **d** Western blot analysis of PHB1 and GRB2 protein levels in EV-treated PBMCs; GAPDH served as a loading control. Band intensities were quantified using ImageJ and normalized to GAPDH, relative values are shown. **e** Schematic of wild-type (WT) and mutant (Mut) PHB1 3′UTR luciferase reporter constructs containing the predicted miR-27a-3p binding site. **f** Relative luciferase activity in HEK293T cells co-transfected with WT or Mut PHB1 3′UTR reporter and miR-27a-3p mimic or negative control. **g** Flow cytometric quantification of human T-cell proliferation (total CD3⁺, CD4⁺, and CD8⁺ subsets) assessed by CFSE dilution after EV treatment, with inhibitor control (IC) or miR-27a-3p inhibitor. **h** Flow cytometric analysis of IFN-γ⁺ Th1, IL-17A⁺ Th17, and CD25⁺FOXP3⁺ Treg populations in human CD4⁺ T cells treated with EVs and/or miR-27a-3p inhibitor. Data are presented as mean ± SD from at least three independent experiments from different human PBMC donors. Technical duplicates were averaged before analysis. Statistical significance was assessed using one-way ANOVA with Tukey’s post hoc test (≥three groups) or paired Student’s t-test (two groups). *p < 0.05, **p < 0.01, ***p < 0.001, n.s.: not significant

To elucidate the molecular mechanisms by which miR-27a-3p-enriched EVs modulate T-cell responses, we first performed target prediction using four independent in silico platforms: TargetScan, miRTarBase, miRDB, and PicTarBase. This integrative analysis identified 18 overlapping candidate genes (Fig. 3a). Among these, PHB1, a mitochondrial chaperone known to regulate T-cell activation and mitochondrial homeostasis, emerged as a top target alongside GRB2, a key adapter protein in the T-cell receptor (TCR) (Fig. 3b). We next validated the predicted targets in activated human peripheral blood mononuclear cells (PBMCs) treated with control or miR-27a-3p-enriched EVs. Quantitative RT-PCR (qRT-PCR) revealed a significant reduction in PHB1 and GRB2 transcript levels in miR-27a-3p-EV-treated cells (Fig. 3c). However, only PHB1 protein expression was consistently downregulated, as confirmed by western blotting (Fig. 3d and Supplementary Fig. 6c), suggesting that PHB1 may be the primary functional target of miR-27a-3p.

To confirm direct interaction, we generated luciferase reporter constructs containing either the wild-type (WT) or mutated (Mut) 3′ untranslated region (UTR) of PHB1. Co-transfection with miR-27a-3p mimics significantly decreased the luciferase activity of the WT construct, whereas the Mut construct lacking the predicted binding site was unaffected (Fig. 3e, f), validating PHB1 as a direct target. Functionally miR-27a-3p inhibition abrogated the immunosuppressive effects of miR-27a-3p-enriched EVs. Carboxyfluorescein succinimidyl ester (CFSE)-based proliferation assays showed that miR-27a-3p inhibition restored the proliferation of total CD3^+^, CD4^+^, and CD8^+^ T cells (Fig. 3g and Supplementary Fig. 6d). Moreover, flow cytometric analysis demonstrated reversal of EV-mediated suppression of Th1 (IFN-γ^+^) and Th17 (IL-17A^+^) cells, along with a reduction in EV-induced expansion of CD25^+^ FOXP3^+^ regulatory T cells (Fig. 3h and Supplementary Fig. 6e). Collectively, these findings indicate that PHB1 is a direct and functional target of miR-27a-3p, and suggest a potential link between EV-delivered miRNA and the suppression of pathogenic T-cell responses in inflammatory settings.

### PD-L1/miR-27a-3p dual-targeting EVs synergistically suppress effector T cells and restrain proximal TCR–Ca²⁺ signaling without inducing apoptosis

**Fig. 4:**
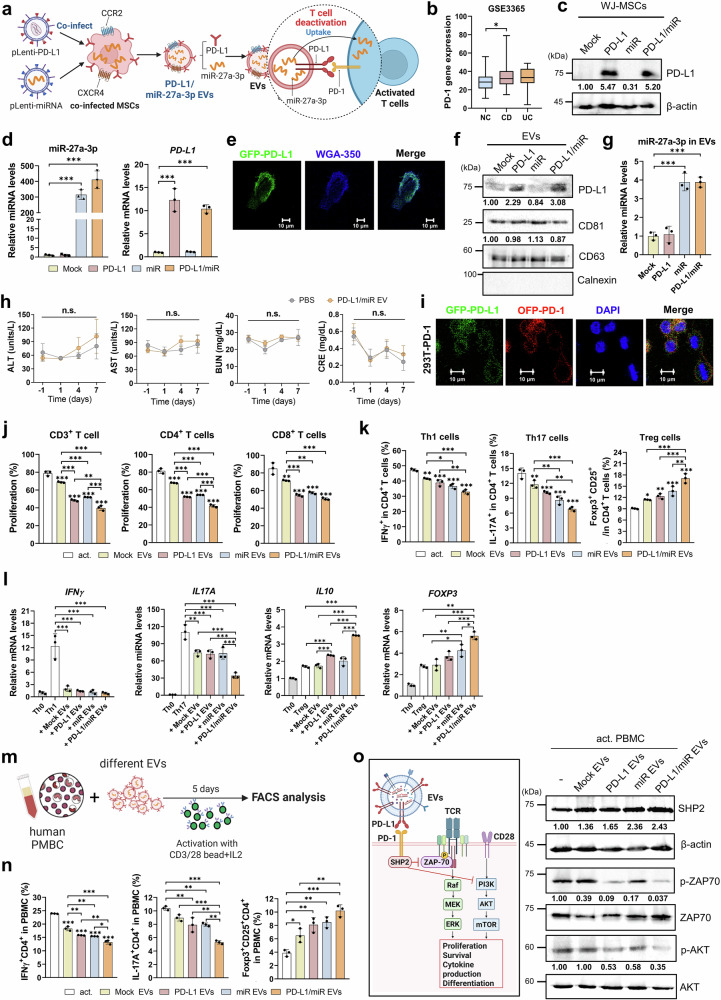
PD-L1-overexpressing, miR-27a-3p-enriched EVs suppress effector T-cell responses and promote FOXP3^+^ Treg induction via PD-1 pathway modulation. **a** Schematic illustrating the generation of engineered EVs from WJ-MSCs co-transduced with lentiviral vectors encoding PD-L1 and miR-27a-3p, followed by isolation of EVs and application to modulate PD-1/PD-L1 interactions in activated T cells (created with BioRender.com). **b** Relative PD-1 expression levels in PBMCs from healthy human donors (normal controls, NC) and patients with Crohn’s disease (CD) and ulcerative colitis (UC), based on the GSE3365 dataset (* *p* < 0.05 vs. healthy (NC). Data were obtained from GSE3365 (Affymetrix Human Genome U133A Array, GPL96). Group sizes: NC *n* = 42, CD *n* = 59, UC *n* = 26. Center lines indicate median. Statistics: one-way ANOVA with Dunnett’s test. **c** Western blot analysis of PD-L1 expression in WJ-MSCs transduced with PD-L1, miR-27a-3p, or PD-L1/miR-27a-3p lentivirus constructs. β-actin was used as a loading control. **d** qRT-PCR analysis of PD-L1 and miR-27a-3p expression in transduced WJ-MSCs. **e** Confocal fluorescence microscopy images showing the localization of GFP-tagged PD-L1 (green) in WJ-MSC membrane stained with WGA-350 (blue). Scale bars**:** 10 μm. **f** Western blot analysis of EV markers (CD63 and CD81) and PD-L1 in isolated EVs. Calnexin was used as a negative control for EV purity. **g** qRT-PCR-based quantification of miR-27a-3p expression in engineered EVs. **h** Serum levels of ALT, AST, BUN, and creatinine following intravenous administration of engineered EVs (500 µg/mouse, day 0); *n* = 5 per group. **i** Confocal microscopy images of EV–cell interactions between GFP-tagged PD-L1 EVs (green) and OFP-tagged PD-1-expressing 293 T cells (red). Nuclei were counterstained with DAPI (blue); scale bar: 10 μm. **j** CFSE-based flow cytometry analysis of human PBMC-derived T cell (total, CD4⁺, and CD8⁺) proliferation following treatment with indicated EVs. **k** Flow cytometric quantification of IFN-γ⁺ Th1, IL-17A⁺ Th17, and CD25⁺FOXP3⁺ Treg subset with human CD4⁺ T cells after EV treatment. **l** Quantitative RT-PCR analysis of IFN-γ, IL-17A, IL-10, and FOXP3 mRNA levels in human CD4⁺ T cells treated with indicated EVs. **m** Schematic workflow of human PBMC stimulation with anti-CD3/CD28 beads and IL-2 in the presence of different EVs. **n** Flow cytometric analysis of CD4⁺ IFN-γ⁺ Th1 cells, CD4⁺ IL-17A⁺ Th17 cells, and CD4⁺ CD25⁺FOXP3⁺ Treg cells in human PBMCs following 5-day culture with the indicated EVs. **o** Western bl**o**t analysis of SHP2, phospho-ZAP70 (p-ZAP70), ZAP70, phospho-AKT (p-AKT), and AKT expression in human CD4^+^ T cells treated with the indicated EVs. β-actin served as a loading control. Schematic diagram of the PD-L1/PD-1 signaling pathway involved in T-cell inhibition (left). Band intensities were quantified using ImageJ and normalized to lane-specific loading controls (cells: β-actin, ZAP70, AKT; EVs: CD63); relative values are shown. Experiments were repeated independently three times with similar outcomes. Data represent mean ± SD from three independent biological replicates, each assessed in duplicate. Statistical significance was assessed using one-way ANOVA with Tukey’s multiple comparisons test. Time-course data (h) were analyzed using two-way repeated-measures ANOVA (group × time) with Sidak’s correction. *p < 0.05, **p < 0.01, ***p < 0.001, n.s.: not significant

To further augment the immunoregulatory efficacy of EVs, we engineered WJ-MSCs to coexpress PD-L1 and miR-27a-3p, thereby generating dual-targeting EVs designed to modulate T cells through both immune checkpoint engagement and miRNA-guided intracellular reprogramming (Fig. 4a). Analysis of the publicly available GSE3365 PBMC transcriptomic dataset revealed significantly elevated PD-1 expression in patients with CD compared with healthy controls, whereas those with UC showed the same increasing trend but did not reach statistical significance (Fig. 4b). Overexpression of PD-L1 and miR-27a-3p in engineered WJ-MSCs was confirmed using qRT-PCR and western blotting (Fig. 4c, d, and Supplementary Fig. 7a). Confocal microscopy revealed proper membrane localization of GFP-tagged PD-L1 (Fig. 4e), and EVs derived from these modified cells retained their characteristic cup-shaped morphology, which was verified using TEM (Supplementary Fig. 7b), and expressed the canonical EV markers CD63 and CD81 without contamination from cellular organelles, as indicated by the absence of calnexin (Fig. 4f and Supplementary Fig. 7c). Western blotting and qRT-PCR analysis confirmed the efficient incorporation of both PD-L1 protein and miR-27a-3p into the EV cargo (Fig. 4f, g and Supplementary Fig. 7c). NTA and zeta potential measurements showed comparable size distribution and surface charges across all the EV preparations (Supplementary Fig. 7d, e). Immunoprecipitation of PD-L1-GFP+ EVs, followed by immunoblotting, further validated the integration of PD-L1 into the EV membrane (Supplementary Fig. 7f). Importantly, systemic administration of PD-L1/miR-27a-3p EVs in mice (500 μg/mouse) revealed no signs of hepatic or renal toxicity, as evidenced by normal levels of ALT, AST, BUN, and creatinine (Fig. 4h), and no histopathological abnormalities in major organs (Supplementary Fig. 8a), confirming the safety profile of dual-targeting EVs.

Investigation of target cell interaction using confocal microscopy revealed specific binding of GFP-PD-L1+ EVs to PD-1-expressing 293 T cells (Fig. 4i), suggesting successful checkpoint engagement. Functional assays demonstrated that PD-L1/miR-27a-3p-enriched EVs suppressed the proliferation of both CD4^+^ and CD8^+^ T cells at significantly greater levels than single-targeting EVs or mock controls (Fig. 4j and Supplementary Fig. 9a). Flow cytometric analysis showed a pronounced reduction in IFN-γ^+^ Th1 and IL-17A^+^ Th17 subsets, alongside a robust expansion of CD25^+^ FOXP3^+^ Tregs (Fig. 4k and Supplementary Fig. 9b). These phenotypic shifts were consistent with transcriptional analysis of stimulated human CD4⁺ T cells, which showed reduced *IFN γ* and *IL17A* and upregulation of *FOXP3* and *IL10*, indicating suppression of effector programs and reinforcement of regulatory pathways (Fig. 4l). In human PBMCs stimulated with anti-CD3/CD28 beads, dual-targeting EVs elicited the most potent immunosuppressive effects in all treatment groups (Fig. 4m, n and Supplementary Fig. 9c). Mechanistically, immunoblot analysis revealed that PD-L1/miR-27a-3p EVs inhibited TCR signaling by reducing the phosphorylation of ZAP70 and AKT, while increasing the expression of SHP2, a key effector in PD-1 signaling (Fig. 4o and Supplementary Fig. 10a). Taken together, these findings demonstrate that dual-targeting EVs synergistically suppress effector T-cell responses by concurrently modulating surface checkpoint pathways and intracellular gene regulatory networks, thereby offering a promising strategy for restoring immune homeostasis in IBD.

We further examined whether these effects involved apoptosis or proximal signaling restraint. Annexin V/7-AAD staining of activated CD4⁺ T cells revealed that PD-L1/miR-27a-3p EVs did not increase apoptosis after 24 or 48 h. In fact, we observed a modest reduction in apoptosis, and this effect was reversed by PD-1 blockade (Supplementary Fig. 10b). Fluo-4 calcium flux assays demonstrated attenuated TCR-induced Ca²⁺ influx in the EV group, which was restored upon PD-1 blockade. These results confirmed the checkpoint-dependent suppression of proximal TCR signaling (Supplementary Fig. 10c).

### PD-L1/miR-27a-3p EVs reprogram inflammatory CD4⁺ T cells by suppressing Th1/Th17 and exhaustion signatures

**Fig. 5:**
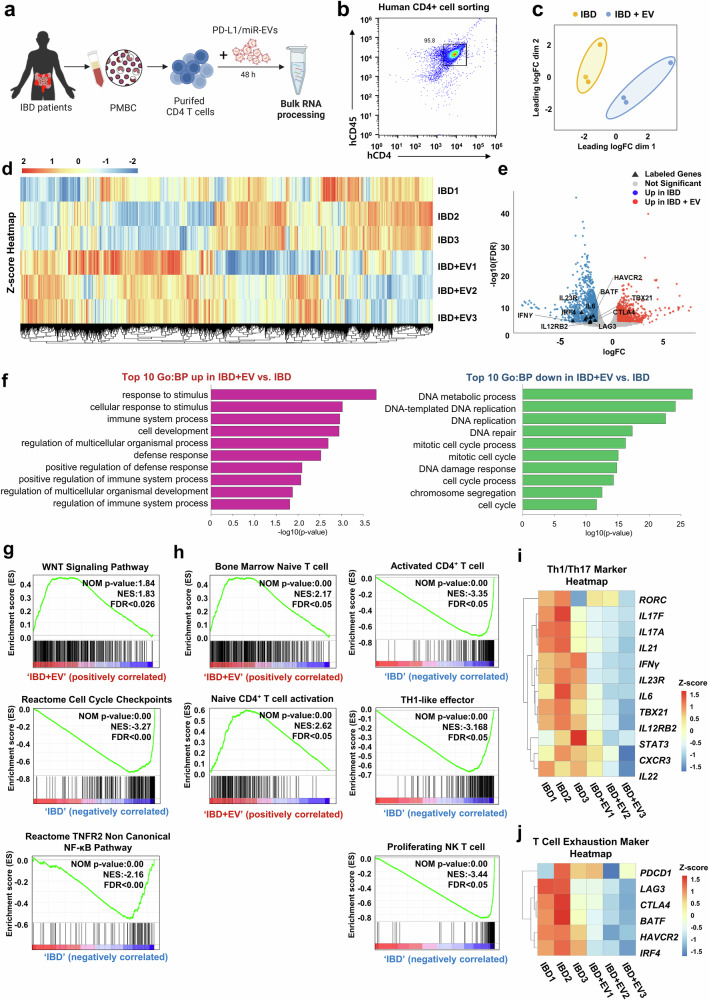
Transcriptomic profiling reveals attenuation of Th1/Th17-related gene signatures in CD4⁺ T cells from patients with IBD following treatment with PD-L1/miR-27a-3p-enriched EVs. **a** Schematic overview of the ex vivo experimental workflow. CD4⁺ T cells were isolated from PBMCs of patients with IBD and treated with PD-L1/miR-27a-3p-enriched EVs for 48 h, followed by bulk RNA sequencing (created with BioRender.com). **b** Representative flow cytometry plot showing >95% purity of human CD4⁺ T cells based on human CD45 and CD4 expression. **c** Multidimensional scaling (MDS) plot showing transcriptomic separation between untreated (IBD) and EV-treated (IBD + EV) CD4⁺ T cells. **d** Heatmap displaying hierarchical clustering of differentially expressed genes (DEGs) between groups (*n* = 3 per group). **e** Volcano plot highlighting significantly upregulated (red) and downregulated (blue) genes in the IBD + EV group compared with the untreated IBD control. Red and blue indicate significantly upregulated and downregulated genes, respectively (FDR < 0.05). **f** Top 10 Gene Ontology biological processes (GO:BP) enriched in upregulated (left) and downregulated (right) DEGs in IBD + EV-treated CD4⁺ T cells (LogFC <0.58). **g** KEGG and Reactome pathway enrichment plots for WNT signaling, cell-cycle checkpoints, TNFR2-NF-κB signaling, and related pathways. **h** Gene set enrichment analysis (GSEA) plots for representative immune-related gene sets, including naive, activated, and Th1 CD4⁺ T-cell signatures. **i**, **j** Heatmap of representative Th1/Th17-related genes (**i**) and T-cell exhaustion-related genes (**j**) across samples; Z-score normalized expression. Bulk RNA-seq data were obtained from three independent patients with IBD (biological replicates)

To explore the impact of dual-targeting EVs on the transcriptional programming of inflammatory T cells in IBD, we performed bulk RNA sequencing on peripheral CD4^+^ T cells isolated from 3 patients with IBD and treated ex vivo with PD-L1/miR-27a-3p-enriched EVs (Fig. 5a and Supplementary Table 1 for patient characteristics). Flow cytometry confirmed that the sorted CD4⁺ T cells from patient-derived PBMCs were highly purified, with >95% CD4⁺ expression (Fig. 5b). Multidimensional scaling (MDS) analysis demonstrated a clear separation between the treated and untreated samples, indicating substantial EV-induced transcriptional reprogramming (Fig. 5c). Hierarchical clustering further supported this distinction, revealing globally altered gene expression profiles (Fig. 5d). Volcano plot analysis identified marked downregulation of proinflammatory Th1/Th17 transcription factors and cytokines, including *IFN γ*, *IL23R, TBX21, BATF*, and *IL12RB2*, in EV-treated CD4^+^ T cells (Fig. 5e). Additionally, exhaustion-related markers, such as *HAVCR2 (TIM-3), LAG3*, and *CTLA4* were significantly decreased. GO enrichment analysis revealed that untreated IBD CD4⁺ T cells were significantly enriched in proliferative programs, including DNA metabolic process, DNA replication, and mitotic cell cycle pathways, consistent with heightened activation. In contrast, EV-treated T cells showed enrichment for immune regulatory and developmental pathways, such as response to stimulus, immune system process, and regulation of multicellular organismal development, indicating a shift toward a more regulated and immunomodulatory transcriptional state (Fig. 5f).

Next, we profiled bulk transcriptomes of patient-derived CD4^+^ T cells treated with PD-L1/miR-27a-3p EVs. Pathway enrichment analyses revealed suppression of cell-cycle checkpoints and TNFR2-NF-κB signaling, accompanied by positive enrichment of WNT signaling (Fig. 5g). EV treatment also reduced proinflammatory cascades, including IL-1, IL-12, interferon, and downstream TCR signaling (Supplementary Fig. 11a). Furthermore, EV-treated cells were positively enriched for the negative regulation of immune effector processes, whereas untreated cells showed enrichment of immune mediator production pathways (Supplementary Fig. 11b). These results are consistent with hallmark-based Gene Set Enrichment Analysis (GSEA), in which EV-treated T cells adopted naive or resting profiles, whereas untreated IBD T cells retained effector-like programs, including Th1- and NK-like signatures (Fig. 5h).

Heatmap visualization corroborated these findings, revealing the coordinated downregulation of *IL17A, IL23R, RORC*, and *TBX21*, which are hallmark genes of Th1 and Th17 lineages (Fig. 5i). Key exhaustion-associated genes, *PDCD1, LAG3*, and *CTLA4*, were also downregulated in the EV-treated group (Fig. 5j). Together, these results demonstrate that PD-L1/miR-27a-3p-enriched induce broad transcriptional changes in inflammatory CD4^+^ T cells by simultaneously suppressing proinflammatory and exhaustion pathways and promoting a homeostatic immune-quiescent state.

### PD-L1/miR-27a-3p EVs preserve epithelial integrity and viability in human intestinal models

**Fig. 6:**
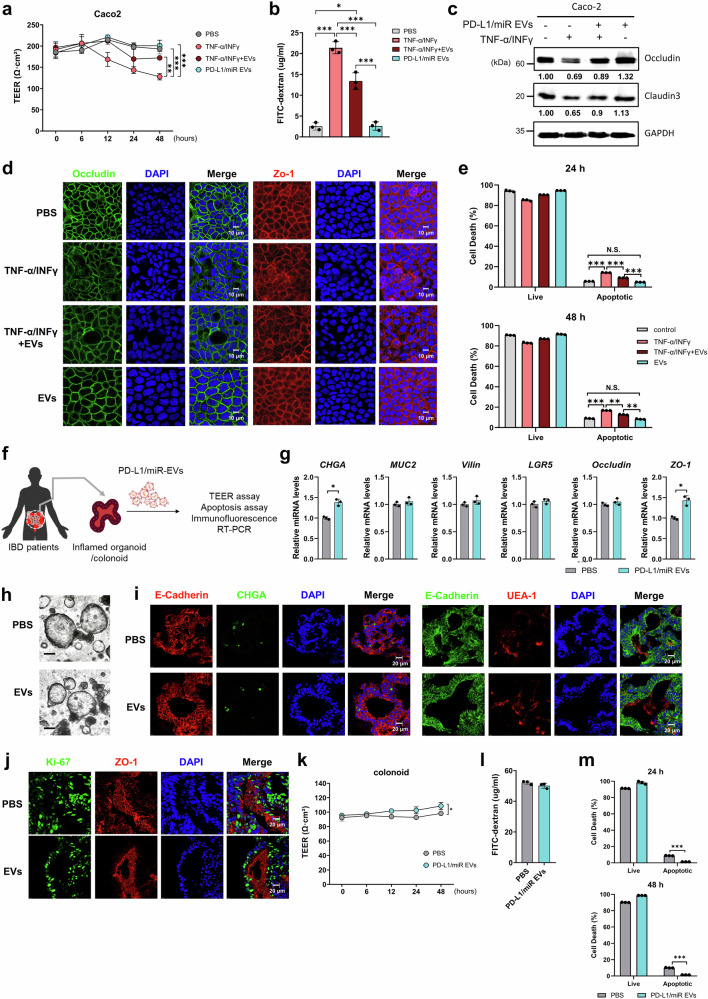
Engineered EVs do not compromise epithelial homeostasis in Caco-2 and patient-derived colonoid monolayers. **a** Transepithelial electrical resistance (TEER) time course (0, 6, 24, and 48 h) in Caco-2 Transwell monolayers cultured (TNF-α/IFN-γ, 10 ng/mL each) with or without PD-L1/miR-27a-3p-enriched EVs (100 μg/mL). Values are area-corrected (Ω·cm²) and normalized to the initial (t = 0 h) resistance of each insert. **b** Paracellular permeability to 4 kDa FITC-dextran at 2 h (apical 1 mg/mL, basolateral sampling; RFU). **c** Immunoblot analysis of occludin, claudin-3, and ZO-1 in Caco-2 monolayers after indicated treatments. GAPDH served as a loading control. Band intensities were quantified using ImageJ and normalized to GAPDH; relative values are shown. **d** Immunofluorescence staining for ZO-1 and occludin in Caco-2 monolayers under basal or cytokine-challenged conditions, with or without EV treatment (scale bar, 10 μm). **e** Flow cytometric analysis of Annexin V/7-AAD staining in Caco-2 cells at 24 and 48 h (live and apoptotic fractions). For Caco-2 experiments, *n* = 3 independent biological replicates with technical triplicates. **f** Schematic illustration of the experimental workflow for treatment of inflamed human 3D colonoids with PD-L1/miR-27a-3p-enriched EVs followed by molecular and functional analyses. **g** qRT-PCR analysis of differentiation and barrier-related genes (CHGA, MUC2, VIL1, LGR5, ZO-1) in colonoids treated with or without EV after differentiation. **h** Representative bright-field images of differentiated colonoids treated with or without EV (scale bar, 100 μm). **i** Representative immunofluorescence images of CHGA, UEA-1 (MUC2), and E-cadherin in differentiated colonoids treated with PBS or PD-L1/miR-27a-3p EVs (scale bar, 20 μm). **j** Immunofluorescence staining of proliferation marker ki67 and tight junction protein ZO-1 in cryosectioned differentiated colonoids after EV treatment (scale bar, 20 μm). **k** TEER time course (0, 6, 24, 48 h) in colonoid-derived 2D Transwell monolayers cultured with or without EVs (100 µg/mL); values are area-corrected and normalized to t = 0 h. **l** FITC-dextran (4 kDa) permeability assay in colonoid-derived monolayers after 2 h incubation (RFU). **m** Annexin V/7-AAD staining in colonoid-derived monolayers at 24 h and 48 h (live and apoptotic fractions). For colonoid experiments, *n* = 3 independent donors with technical triplicates. Data are presented as mean ± SD with individual data points shown. TEER courses were analyzed at the 48-h endpoint using one-way ANOVA with Dunnett’s correction (comparison vs baseline or cytokine-only, as specified). Single time-point assays (FITC-dextran, IF quantification, apoptosis) were analyzed using one-way ANOVA with Sidak’s correction or paired t-test as indicated. Significance: *p < 0.05, **p < 0.01, ***p < 0.001

To assess whether dual-targeting EVs affect epithelial integrity or viability, we first examined barrier function and junctional organization in Caco-2 monolayers. PD-L1/miR-27a-3p EVs did not alter baseline transepithelial electrical resistance (TEER) or paracellular permeability (Fig. 6a, b). Under TNF-α/IFN-γ challenge, EV treatment partially preserved TEER and reduced FITC-dextran leakage, indicating the maintenance of barrier function. Immunoblotting and immunofluorescence analyses showed preserved expression and localization of occludin, claudin-3, and ZO-1 with partial recovery under inflammatory stress (Fig. 6c, d, and Supplementary Fig. 12a). Flow cytometric Annexin V/7-AAD analysis confirmed that there was no significant increase in apoptosis after 24 or 48 h (Fig. 6e and Supplementary Fig. 12b).

Next, human colonoids derived from IBD patients were used to validate epithelial compatibility of EVs under physiological conditions (Fig. 6f). qRT-PCR analysis revealed that EV treatment did not significantly alter the expression of differentiation or barrier-related genes, including *MUC2*, *VIL1*, *LGR5*, and *ZO-1* (Fig. 6g). Representative bright-field images showed preserved morphology and lumen architecture in EV-treated colonoids comparable to PBS controls (Fig. 6h). Immunofluorescence staining confirmed maintenance of epithelial and lineage markers, including E-cadherin, CHGA, and UEA-1, indicating intact differentiation and barrier organization (Fig. 6i). Similarly, Ki-67 and ZO-1 co-staining demonstrated preserved proliferative capacity and junctional integrity following EV exposure (Fig. 6j). In colonoid-derived 2D Transwell monolayers, EVs maintained TEER and paracellular permeability comparable to controls (Fig. 6k, l), and no increase in apoptosis was detected at either 24 h or 48 h (Fig. 6m and Supplementary Fig. 12c). Collectively, these data indicate that PD-L1/miR-27a-3p-enriched EVs preserve epithelial viability, differentiation, and barrier integrity without inducing cytotoxic or off-target effects in human intestinal epithelial models.

### PD-L1/miR-27a-3p-enriched EVs alleviate intestinal inflammation in a humanized colitis model

To evaluate the therapeutic efficacy of dual-targeting EVs in vivo, we established a humanized colitis mouse model in which NSG mice were adoptively transferred with human PBMCs or CD4^+^ T cells, followed by induction of colitis using trinitrobenzene sulfonic acid (TNBS) (Supplementary Fig. 13a). Mice engrafted with human PBMCs developed classic features of intestinal inflammation, including significant body weight loss, an elevated DAI, colonic shortening, and increased human CD45^+^ leukocyte infiltration in the peripheral blood and spleen (Supplementary Fig. 13b–e). Histological and immunohistochemical analyses further revealed marked infiltration of CD3^+^ T cells and enhanced colonic expression of *TNFα, IL-1β*, and *PD-1* (Supplementary Fig. 13f–h).

**Fig. 7:**
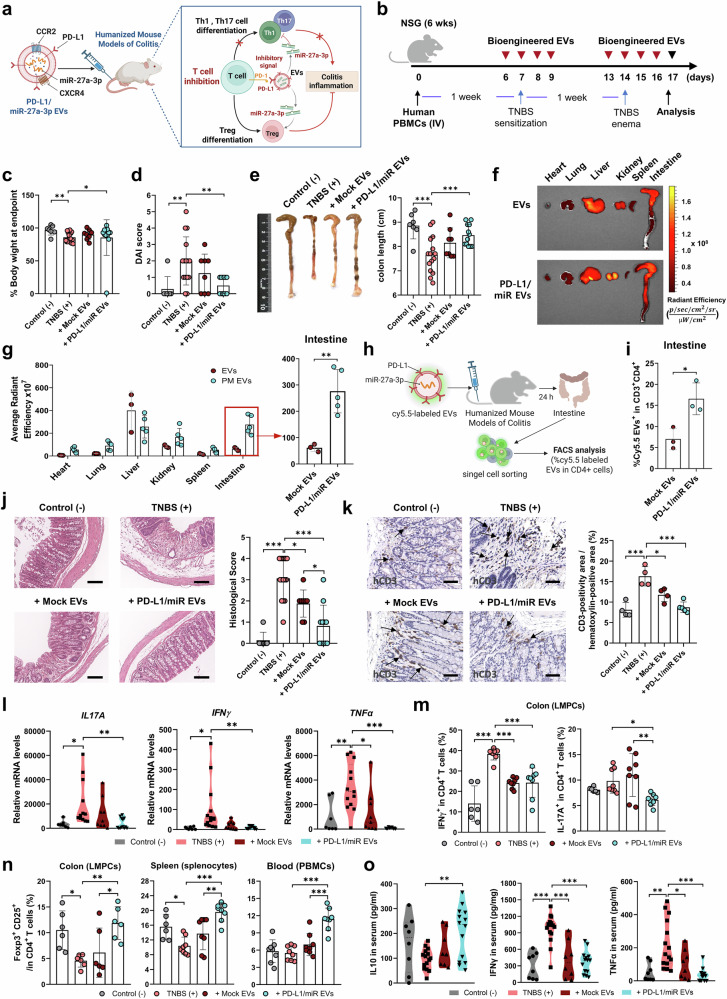
PD-L1/miR-27a-3p-enriched EVs restore Th17/Treg balance and attenuate T cell-driven inflammation in a humanized colitis model. **a** Schematic illustration of immunomodulatory mechanism of PD-L1/miR-27a-3p EVs in a humanized mice with TNBS-induced colitis (created with BioRender.com). **b** Experimental timeline showing human PBMC engraftment into NSG mice, followed by rectal TNBS administration and intravenous EV treatment over 2 weeks. **c** Body weight changes (%) monitored over 3 days following TNBS administration in Control, TNBS alone, TNBS + Mock EV, and TNBS + PD-L1/miR EV-treated mice. **d** DAI scores assessed 3 days after TNBS enema. **e** Representative colon images and quantification of colon length on day 17 post-colitis induction. **f** Representative IVIS fluorescence images showing biodistribution of Cy5.5-labeled Mock EVs versus PD-L1/miR EVs in major organs, including the intestine. **g** Quantitative analysis of IVIS signal intensity in explanted organs; red box highlights EV localization in the intestine. **h** Schematic workflow for isolation of colon lamina propria cells and detection of EV uptake (created with BioRender.com). **i** Flow cytometry analysis of Cy5.5-labeled EV uptake in colonic human CD4⁺ T cells. **j** Representative H&E-stained colon sections and corresponding histopathological scores evaluating inflammation severity (Scale bars: 100 μm). **k** Representative immunohistochemical images of human CD3^+^ T-cell (black arrows) infiltration in colonic tissue across experimental groups; CD3⁺ T-cell densities were quantified in each group (Scale bars: 50 μm). **l** qRT-PCR analysis of human *IL17A, IFN- γ*, and *TNF-α* mRNA levels in colonic tissues from each experimental group. **m** Flow cytometric quantification of human IFN-γ⁺ Th1 and IL-17A⁺ Th17 cells among colonic human CD4⁺ lamina propria mononuclear cells (LPMCs) from the colon. **n** Flow cytometric analysis of CD25⁺FOXP3⁺ Treg cells in human CD4⁺ T cells isolated from colon, spleen, and peripheral blood. **o** Serum levels of IL-10, IFN-γ, and TNF-α in each group, measured using ELISA. Data are presented as mean ± SD. Each group included 7–15 mice (biological replicates), and all measurements were performed in duplicate and averaged before analysis. Cytokine ELISA and qRT-PCR were measured in triplicate technical replicates. Statistical significance was assessed using one-way ANOVA with Tukey’s multiple comparisons test or unpaired Student’s t-test. *p < 0.05, **p < 0.01, ***p < 0.001

Systemic administration of PD-L1/miR-27a-3p-enriched EVs, initiated either before or after the TNBS challenge, markedly improved colitis outcomes (Fig. 7a, b). In the TNBS colitis model, treatment with engineered EVs improved early body weight and DAI trajectories (days 1–3) and produced significantly less weight loss, lower DAI scores, and longer colons than the mock-EV controls (Fig. 7c–e and Supplementary Fig. 14a, b). Biodistribution analyses using Cy5.5-labeled EVs revealed preferential accumulation of WJ-MSC-derived EVs in the intestine, compared with 293T-EVs, potentially owing to higher CCR2 and CXCR4 expression in WJ-MSCs (Fig. 1b and Supplementary Fig. 14d, e). In vivo imaging system (IVIS) tracking and flow cytometry confirmed enhanced localization of PD-L1/miR-27a-3p EVs in the inflamed intestine and their uptake by CD4^+^ T cells (Fig. 7f–i). Histopathological evaluation further demonstrated improved mucosal integrity and reduced inflammatory cell infiltration in the colon after treatment with EVs (Fig. 7j).

### PD-L1/miR-27a-3p EVs suppress intestinal Th1/Th17 responses and promote regulatory immunity

Next, we investigated the immunological mechanisms underlying the EV-mediated protection. Immunohistochemical analysis revealed a marked reduction in CD3^+^ T-cell infiltration in the colonic lamina propria of PD-L1/miR-27a-3p EV-treated mice, indicating effective suppression of mucosal T-cell recruitment (Fig. 7k). PD-L1/miR-27a-3p EVs showed the lowest CD3⁺ density among groups. Colonic mRNA levels of *IL-17A, IFNγ*, and *TNFα* were markedly reduced relative to TNBS controls (Fig. 7l). Flow cytometric analysis of colonic lamina propria mononuclear cells (LPMCs), splenocytes, and PBMCs demonstrated that PD-L1/miR-27a-3p EV–treated mice showed significantly lower frequencies of IFN-γ⁺ Th1 and IL-17A⁺ Th17 cells, accompanied by a concurrent increase in CD25⁺FOXP3⁺ regulatory T cells (Fig. 7m, n and Supplementary Fig. 15a–c). These cellular changes were accompanied by systemic immunomodulation, as serum levels of IL-10 were significantly elevated, whereas those of IFN-γ and TNF-α were reduced in EV-treated mice (Fig. 7o). Although not all comparisons between mock EVs and PD-L1/miR-27a-3p EVs reached statistical significance, the direction and magnitude of immunomodulation were concordant across histological, transcriptional, and flow-cytometric readouts. These data indicate that PD-L1/miR-27a-3p-enriched EVs not only attenuate pathogenic T-cell responses, but also promote the induction of regulatory immunity, thereby contributing to the resolution of intestinal inflammation.

### Single-cell transcriptomics revealed that PD-L1/miR-27a-3p EVs reprogram CD4⁺ T cells toward immunoregulatory states

**Fig. 8:**
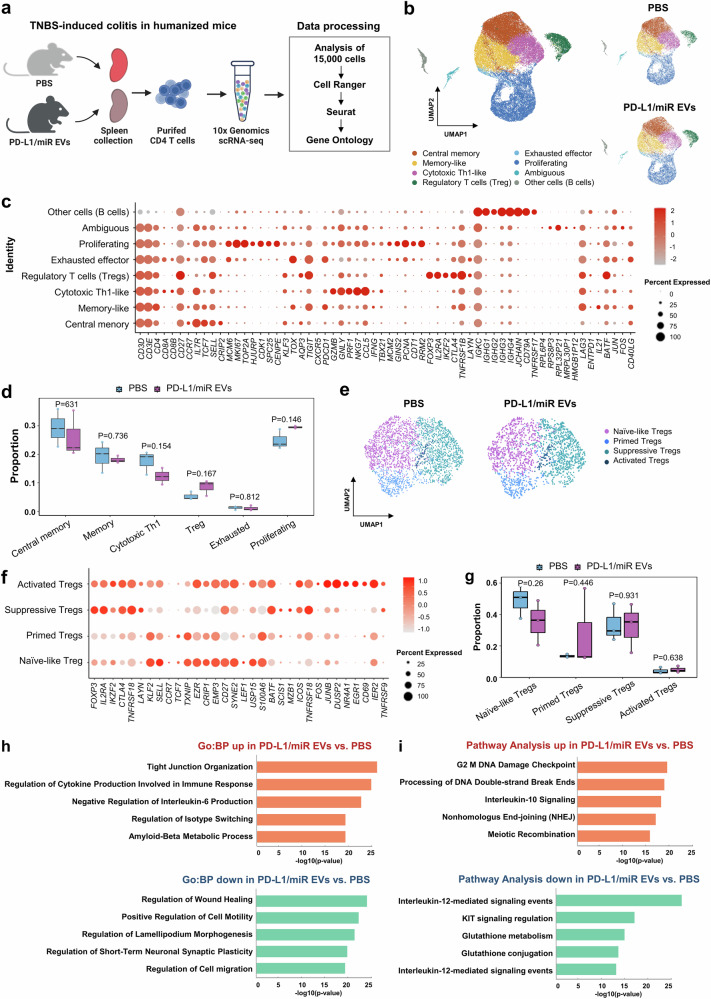
Single-cell transcriptomics of purified human CD4⁺ T cells from humanized mice models of colitis reveals Treg program modulation by PD-L1/miR-27a-3p EVs. **a** Schematic illustration of the single-cell RNA sequencing workflow. Human CD4⁺ T cells were isolated from the spleens of humanized mice models of colitis treated with PBS or PD-L1/miR-27a-3p EVs and subjected to 10x Genomics-based scRNA-seq. This dataset was intentionally restricted to purified CD4⁺ T cells (created with BioRender.com). **b** UMAP visualization of human CD4⁺ T-cell clusters in each treatment group. **c** Dot plot showing the expression of representative marker genes used for human CD4⁺ T-cell subset annotation. Dot size indicates the proportion of expressing cells, and color intensity reflects the average expression level. Minor non-T clusters (<2%; Ambiguous, Other cells (B cells)) emerged after unsupervised clustering and are displayed in (**b**, **c**) for completeness. **d** Boxplot comparing the proportions of annotated human CD4⁺ T-cell subsets between the PBS- and EV-treated groups. **e** UMAP plots showing the distribution of FOXP3⁺ Treg cluster in each group. **f** Dot plots showing expression profiles of key genes across Treg subclusters, including naive-like, primed, suppressive, and activated Tregs. **g** Boxplots comparing the relative frequency of each Treg subcluster between the groups. **h** GO enrichment analysis of DEGs in Tregs, showing enriched biological processes for upregulated genes (orange) and downregulated genes (green) in the EV-treated group. **i** Pathway enrichment analysis of Treg transcriptomes from PBS- and EV-treated groups. Data represent pooled splenic CD4⁺ T cells from the spleens of three human donors per group (biological replicates). Each group was analyzed as one scRNA-seq library. Statistical comparisons were performed using unpaired two-tailed Student’s t-test. *p*-values are indicated in each panel, and no comparisons reached statistical significance. Data are presented as boxplots showing the median, interquartile range (IQR), and full range

To further characterize how PD-L1/miR-27a-3p-enriched EVs modulate CD4^+^ T-cell responses, we performed single-cell RNA sequencing (scRNA-seq) on splenic CD4^+^ T cells isolated from humanized mice models of colitis treated with either PBS or dual-targeting EVs (Fig. 8a). High-purity human CD4^+^ T cells were obtained through magnetic enrichment and FACS sorting (>90% purity; Supplementary Fig. 16a), and transcriptomes from 22,266 PBS-treated and 21,896 EV-treated cells were profiled using the 10x Genomics platform (Supplementary Table 2).

Unsupervised clustering identified eight major human CD4⁺ T-cell populations—central memory, memory-like, cytotoxic Th1-like, regulatory T cells (Tregs), exhausted effectors, and proliferating cells, and two minor subsets: ambiguous and B cell–like clusters (Fig. 8b). Cluster annotations were guided by canonical marker genes such as *IL7R* (memory), *GZMB* (cytotoxic), *FOXP3* (Treg), and *PDCD1* (exhaustion), and cell cycle–associated genes including *MCM6, CDK1*, and *RPL32P21* (Fig. 8c and Supplementary Fig. 16b), consistent with established single-cell studies that delineated CD4⁺ T-cell subsets in both homeostatic and inflammatory conditions.^27–29^ Using the FindAllMarkers algorithm, we identified defining transcripts for each cluster, enabling comparative analysis of EV-treated versus control samples (Supplementary Table 3). Although statistical significance was limited by sample variability, EV-treated samples exhibited consistent shifts in subset distribution, with increased frequencies of FOXP3^+^ Tregs and proliferating CD4⁺ T cells and reduced proportions of central memory and cytotoxic Th1-like subsets relative to PBS controls (Fig. 8d and Supplementary Fig. 16c and Supplementary Table 4). These shifts suggest an EV-mediated bias toward regulatory and reparative phenotypes.

Subclustering analysis focused on the Treg compartment resolved four transcriptionally distinct FOXP3⁺ subsets: naive-like, primed, suppressive, and activated Tregs (Fig. 8e and Supplementary Fig. 16d, e and Supplementary Table 5). Suppressive and activated Tregs from EV-treated mice expressed elevated levels of *FOXP3, CTLA4*, *IL2RA, JUNB*, and *TNFRSF9*, consistent with an immunoregulatory phenotype previously linked to enhanced suppressive function in Tregs,^30^ whereas naive-like and primed subsets exhibited high expression of *SELL, TCF7*, *LEF1*, *KLF2*, and *CCR7* (Fig. 8f and Supplementary Fig. 16f).^31^ Boxplot analysis suggested a modest enrichment of suppressive Tregs in the EV group, accompanied by a relative decline in primed and naive-like subsets (Fig. 8g) To gain functional insights, we performed GO enrichment analysis of differentially expressed genes within the Treg compartment (Supplementary Table 6). EV-treated Tregs showed significant enrichment of pathways involved in tight junction assembly, negative regulation of IL-6 production, and cytokine-mediated immune responses (Fig. 8h). Hallmark pathway analysis further revealed upregulation of IL-10 signaling, G2/M checkpoint control, and non-homologous end-joining (NHEJ) DNA repair, suggesting enhanced epithelial protection and stress resilience (Fig. 8i). Conversely, Tregs from PBS-treated mice were enriched in pathways associated with wound healing, migration, IL-12 signaling, and glutathione metabolism, reflecting a more inflammatory and Th1-skewed profile (Fig. 8h, i). Altogether, these single-cell transcriptomic data indicate that PD-L1/miR-27a-3p-enriched EVs reshape the CD4^+^ T-cell landscape by promoting regulatory subsets and rewiring gene programs toward immune tolerance and tissue homeostasis.

## Discussion

**Fig. 9:**
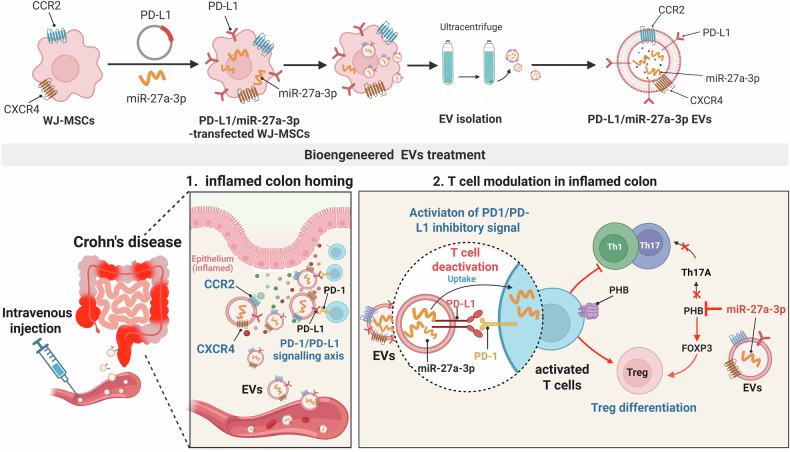
Graphical summary of dual-targeting EV immunotherapy. Engineered WJ-MSC-derived EVs display PD-L1 on the surface and encapsulate miR-27a-3p, thereby enabling the modulation of CD4^+^ T cells in IBD. The platform facilitates homing to inflamed tissues, checkpoint engagement, metabolic suppression of Th17 cells, and expansion of regulatory T cells. Graphical summary was created using BioRender

IBD remains a therapeutic challenge characterized by persistent mucosal inflammation and dysregulated T-cell responses that are not adequately controlled by conventional advanced therapies.^32^ Although immune checkpoint inhibitors have revolutionized cancer therapy, their direct application in autoimmune and chronic inflammatory diseases such as IBD is limited because of systemic immune activation and poor tissue selectivity.^33,34^ We addressed these challenges using a dual-functional EV platform that synchronizes PD-L1-mediated checkpoint engagement with miR-27a-3p-driven reprogramming of T-cell phenotypes, as summarized in Fig. 9.

Mechanistically, PD-L1 displayed on the EV surface inhibited TCR signaling through activation of SHP2 and subsequent inhibition of AKT phosphorylation,^35,36^ whereas miR-27a-3p targeted PHB1, a mitochondrial scaffold protein involved in Th17 metabolism.^9,11^ Although PHB1 is known for its role in maintaining mitochondrial homeostasis in T cells, our findings identify a previously unrecognized immunometabolic checkpoint function modulating the balance between Th17 and FOXP3⁺ Treg differentiation in human CD4⁺ T cells. Consistent with this mechanism, PD-L1/miR-27a-3p EVs dampened TCR proximal calcium signaling and reduced effector cytokine output without inducing apoptosis, confirming that the suppression was checkpoint-dependent rather than cytotoxic.

Unlike previously studied miRNAs, such as proinflammatory miR-21 and miR-155 that drive mucosal damage^37,38^ or the anti-inflammatory miR-146a and miR-200 family that have shown only modest effects in colitis models,^39,40^ the engineered EV-mediated delivery of miR-27a-3p used in this study achieves robust and localized immune reprogramming in a cell-free format. Recent clinical progress with obefazimod, a miR-124 inducer currently in late-phase clinical trials for UC, further supports the translational potential of miRNA-based immune modulation strategies in IBD.^13^ Integrated transcriptomic analyses revealed a coordinated reprogramming of inflammatory T-cell states. We consistently observed a shift from effector memory to regulatory phenotypes across both bulk and single-cell datasets. Although the single-cell analysis strongly captured Treg induction and Th1 suppression, no statistically significant decrease in the Th17 cluster was detected. This likely reflects the inherent plasticity of Th17 cells and the limited sensitivity of scRNA-seq for relatively rare IL-17A+ subsets.^5^ Nevertheless, qRT-PCR and flow cytometry confirmed the suppression of Th17-associated functions, reinforcing the overall conclusion of coordinated T-cell reprogramming. Importantly, similar transcriptomic shifts were observed in ex vivo-stimulated CD4^+^ T cells from patients with IBD, suggesting that subclinical immune dysregulation persists and could be amenable to therapeutic modulation using our approach. Although PD-L1/miR-27a-3p EVs consistently exhibited stronger anti-inflammatory trends than mock EVs, some comparisons did not reach statistical significance. This variability likely reflects both the intrinsic heterogeneity of the colitis model and the baseline immunoregulatory activity of unmodified MSC-derived EVs (mock EVs). Nevertheless, the concordant reductions in Th1/Th17 responses and the enhancement of Treg-associated markers indicate a biologically meaningful reinforcement of immune regulation by PD-L1/miR-27a-3p EVs.

In PBMCs, PD-1 expression was significantly increased in CD, whereas UC showed a non-significant increasing trend. In contrast, mucosal studies have consistently reported significantly higher PD-1/PD-L1 expression in UC, underscoring compartment-specific checkpoint activation.^41,42^ Disease-specific nuances may influence the magnitude of response; however, as our approach directly targets CD4⁺ T-cell dysregulation, which is a shared hallmark of CD and UC, it is expected to confer therapeutic benefits across IBD. Compared with biologics that neutralize individual cytokines, such as TNF-α or IL-23,^43,44^ the PD-L1/miR-27a-3p EVs used in this study offer multi-layered immunomodulation through checkpoint signaling, miRNA-driven reprogramming, and restoration of regulatory networks in a scalable, low-toxicity format. Our findings extend and refine prior efforts to use MSCs and MSC-EVs in IBD therapy. Although MSC-based treatments have demonstrated clinical efficacy, particularly in perianal fistulizing CD, the ability of transplanted MSCs to engraft at the sites of inflammation is limited, often resulting in suboptimal cell numbers for effective immunomodulation. Furthermore, the safety profile of MSC therapy remains to be fully elucidated, with concerns over potential immunogenicity, unintended differentiation, and tumorigenicity.^45^ In contrast, treatment with MSC-derived EVs can mitigate the safety and immunogenicity concerns while preserving the essential immunomodulatory properties.^46^ The engineered EVs used in this study further enhance this therapeutic modality by incorporating defined immune-targeting elements (PD-L1 and miR-27a-3p), thereby enabling dual checkpoint and metabolic modulation in a tissue-accessible, scalable, and customizable format. Moreover, the synchronization of surface checkpoint signaling with intracellular metabolic control may extend the utility of this platform to other T-cell-driven disorders such as autoimmune hepatitis, type 1 diabetes, or inflammatory arthritis.^34,47,48^

Despite these advantages, some limitations remain. Although the EVs preferentially localize to the inflamed intestine, biodistribution imaging revealed their localization in the liver and kidneys, indicating that gut targeting is not exclusive. Cross-organ safety assessments, including histology, serum chemistry, and clinical monitoring showed no toxicity at the tested dose; however, further optimization of the targeting moieties and administration parameters is important to reduce extraintestinal exposure. Additionally, although PHB1 was identified as a key target of miR-27a-3p, the downstream molecular circuitry governing FOXP3⁺ Treg differentiation and long-term stability require further study, potentially through targeted perturbation and epigenetic profiling. Finally, although our humanized mouse model recapitulates the key features of IBD, future integration of patient-derived organoid-immune co-cultures and spatial transcriptomics will enhance translational fidelity. In summary, this study introduces a next-generation EV-based platform that combines surface checkpoint signaling with miRNA-guided metabolic reprogramming to restore mucosal immune tolerance. By integrating spatial targeting and dual-pathway modulation, our system offers a durable, cell-free strategy for IBD and other T cell-driven inflammatory disorders.

## Materials and methods

### Study approval

All human and animal experiments were conducted in accordance with ethical guidelines and approved by the appropriate institutional committees. Peripheral blood samples from healthy donors were obtained with the approval of the Institutional Review Board of Seoul National University (IRB No. E2309/002-00), patient-derived PBMCs were collected with IRB approval from Seoul National University College of Medicine and Seoul National University Hospital (IRB No. 2012-115-1183), and mucosal pinch biopsies of ascending colon from 3 patients with UC were collected during using 2.8-mm standard biopsy forceps with IRB of New York University Grossman School of Medicine (Mucosal Immune Profiling in Patients with Inflammatory Bowel Disease; IRB No. S12-01137), with written informed consent obtained from all participants. All animal study protocols were approved by the Institutional Animal Care and Use Committee of Seoul National University (IACUC No. SNU-230918-1-2).

### In vitro T cell assays

Human PBMCs were isolated using density gradient centrifugation (Ficoll-Paque PLUS; GE Healthcare) and activated with anti-CD3/CD28 Dynabeads in the presence of IL-2 (30 U/mL) for 5 days, during which EVs were concurrently administered.^49^ Human CD4⁺ T cells were purified using magnetic-activated cell sorting (MACS; Miltenyi Biotec, Bergisch Gladbach, Germany) and treated with EVs for 6 days. For proliferation assays, cells were labeled with carboxyfluorescein succinimidyl ester (CFSE; Thermo Fisher Scientific, Waltham, MA, USA) prior to treatment with EVs. For polarization, purified human CD4⁺ T cells were stimulated with anti-CD3/CD28 microbeads plus IL-2 (20 U/mL) and culturing for 5 days in the presence of EVs under lineage-specific conditions: Th1 was induced with IFN-γ (25 ng/mL) and IL-12 (25 ng/mL); Th17 with IL-6 (50 ng/mL) and TGF-β (25 ng/mL); and Treg with TGF-β (25 ng/mL) and all-trans-retinoic acid (10 nM). For intracellular cytokine staining, the cells were restimulated with a cell-stimulation cocktail (PMA/ionomycin with transport inhibitors; Invitrogen) for 5 h before fixation. FOXP3/transcription-factor staining was performed using a Foxp3/Transcription Factor Staining Buffer Set (eBioscience). The following flow cytometry antibodies (fluorochrome-conjugated) were used: CD3 (HIT3a), CD4 (RPA-T4), CD8 (RPA-T8), CD45 (HI30), IFN-γ (4S. B3), IL-17A (SCPL1362), CD25 (M-A251), and FOXP3 (MF23) (BD Biosciences). Cell viability was assessed using the 7AAD assay.

### DSS-induced colitis model

Male C57BL/6 mice were provided drinking water containing 3% DSS for 7 days. EVs were administered intraperitoneally on days 1, 3, and 5, corresponding to a total dose of 300 μg per mouse. Mice were sacrificed on day 10 for evaluation of colon length, histopathology, MPO activity, and DAI. DAI was recorded daily as the sum of weight loss, stool consistency, and fecal blood scores (0–4 each; total 0–12) according to the information in Supplementary Table 7.

### Mechanistic validation of miR-27a-3p targeting

Synthetic miR-27a-3p mimics or inhibitors were transfected into WJ-MSCs or CD4⁺ T cells using Lipofectamine RNAiMAX. Dual-luciferase reporter assays were performed using PHB1 3′UTR constructs to confirm direct targeting.

### Dual genetic engineering of EVs

Lentiviral vectors encoding hsa-miR-27a-3p (Abm, Richmond, Canada) and human PD-L1 (Sino Biological, Beijing, China) were introduced into WJ-MSCs by coinfection with viral supernatants from HEK293T cells. Transduced cells were selected with puromycin (2 µg/mL) and sorted using FACS to isolate GFP⁺ populations. Expression of PD-L1 and miR-27a-3p in EVs was confirmed using western blotting and qRT-PCR.

### Patient-derived colonoid culture and monolayers

Colonoids derived from patients with UC were embedded in Matrigel (30 µL) and maintained in Human IntestiCult™ Organoid Growth Medium (STEMCELL Technologies, Seattle, WA) supplemented with antibiotics.^50^ For passaging, Y-27632 (10 µM) was added for 3 days. Differentiation was induced using a 1:1 mixture of IntestiCult Basal Medium and DMEM/F-12 with supplements for 4 days.

For monolayers, dissociated colonoids were seeded on Matrigel-coated plates or Transwells (1 × 10⁵ cells/well) and differentiated for 4 days until TEER stabilized. EVs (100 µg/mL) were applied apically for 48 h, and TEER, FITC-dextran permeability, and Annexin V/7-AAD assays were performed as described for Caco-2 monolayers.

### Colonoid immunofluorescence

Fixed organoids were cryoprotected in 30% sucrose, embedded in a gelatin/sucrose matrix, and cryosectioned at 14-µm thickness. Sections were stained for lineage and junctional markers, including UEA-1 (Vector Laboratories, Burlingame, CA, USA), CHGA (Abcam, Cambridge, UK), Ki-67 (DAKO), ZO-1 (Affinity Biosciences), and E-cadherin (Santa Cruz Biotechnology, Dallas, TX, USA) and counterstained with DAPI.

### Bulk RNA sequencing of CD4⁺ T cells treated with PD-L1/miR-27a-3p EVs

CD4⁺ T cells from PBMCs of patients with IBD were treated with PD-L1/miR-27a-3p EVs for 48 h. Total RNA was extracted and sequenced as described earlier. Differential expression was analyzed using edgeR (v3.32.1) with a cutoff of |log2FC| ≥ 0.58 and FDR < 0.05. Pathway analysis included GO and Gene Set Enrichment Analysis (GSEA).

### TNBS-induced colitis in humanized mice

NOD-scid-IL2Rγc⁻/⁻ (NSG) mice were intravenously injected with 2 × 10⁷ human PBMCs and allowed to engraft for 7 days. To induce colitis, mice were first sensitized by subcutaneous injection of 100 μL of 1% TNBS (2,4,6-trinitrobenzenesulfonic acid, Sigma-Aldrich) dissolved in PBS. On day 14, colitis was induced via rectal administration of 2.5% TNBS in 50% ethanol (100 μL total volume) under isoflurane anesthesia. EVs (a total dose of 400 μg per mouse) were administered intravenously during both preventive (days 6–9) and therapeutic (days 13–17) periods. The mice were monitored daily for body weight loss and clinical symptoms, and the DAI was calculated as the sum of the scores for body weight change, activity/posture, and stool/anal condition (0–4 each; total 0–12), as described in Supplementary Table 8. On day 17, colon tissues were harvested for histopathological, immunohistochemical (IHC), and flow cytometry analyses.

### Single-cell capture, library preparation and sequencing

Single-cell RNA sequencing (scRNA-seq) was performed using human CD4⁺ T cells isolated from the spleens of humanized mice models of colitis. NSG mice were reconstituted with human PBMCs from three independent donors and treated with either PBS or PD-L1/miR-27a-3p-enriched EVs. Human CD4⁺ T cells were isolated from the spleens and pooled by treatment groups across donors to generate one scRNA-seq library per condition. Cells were counted using a LUNA-FL Automated Fluorescence Cell Counter (Logos Biosystems, Anyang, Korea). Libraries were constructed using the Chromium Controller and Chromium Next GEM Single Cell 3’ Reagent Kit v3.1 (10x Genomics, Pleasanton, CA, USA) according to the manufacturer’s protocol. Single cells were encapsulated into Gel Bead-in-Emulsions (GEMs), and barcoded cDNA was synthesized, amplified, and processed through fragmentation, end-repair, A-tailing, adapter ligation, and index PCR to generate 3’ gene expression libraries. The final libraries were purified and quantified using qPCR (KAPA Biosystems, Wilmington, MA, USA), and quality-checked using the 4200 TapeStation system (Agilent Technologies, Santa Clara, CA, USA). Sequencing was performed on an Illumina HiSeq platform (Illumina, San Diego, CA, USA) using 150 bp paired-end reads with an average depth of approximately 40,000 reads per cell.

### Statistical analysis

All data are presented as mean ± standard deviation (SD). Statistical comparisons were performed using unpaired or paired two-tailed Student’s t-tests or one-way ANOVA, followed by Sidak’s, Dunnett’s, or Tukey’s multiple comparisons tests, as appropriate. Time-course data were analyzed using two-way repeated-measures ANOVA (group × time) with Sidak’s correction. Statistical significance was set at *p* < 0.05.

n denotes the number of independent biological replicates (individual mice or human donors). For experiments with technical replicates (qPCR, ELISA, and flow cytometry), replicate values were averaged for each biological replicate before statistical testing. The specific statistical tests and replicate numbers used in each experiment are detailed in the corresponding figure legends. All analyses were performed using GraphPad Prism (v9.0; GraphPad Software, San Diego, CA, USA).

## Supplementary information

Revised Supplementary Materials

Supplementary Tables 3

Supplementary Tables 5

Supplementary Tables 6

## Data Availability

The RNA-seq datasets generated in this study have been deposited in the NCBI Gene Expression Omnibus (GEO) under the accession numbers GSE302696, GSE302709, and GSE302712, and are publicly available.
