# Supplementary material for: Engineered extracellular vesicles reprogram T cells by targeting PD-1 and PHB1 signaling in inflammatory bowel disease
Source: Signal Transduct Target Ther. 2025 Dec 25;10:418. doi: 10.1038/s41392-025-02516-0 (PMC12738735; doi:10.1038/s41392-025-02516-0)

Supplementary Materials for

**Engineered Extracellular Vesicles Reprogram T Cells by Targeting PD-1 and PHB1 Signaling in Inflammatory Bowel Disease**

Mi-Kyung Oh^1^, Hyun Sung Park^1,2^, Dong-Hoon Chae^1^, Aaron Yu^1^, Jae Han Park^1^, Jiyoung Heo^1^, Keonwoo Cho^1^, Jiho Kim^1^, Byeonghwi Lim^3^, Jun-Mo Kim^3^, Jordan E. Axelrad^4^, Kyung Ku Jang^5^, Jong Pil Im^6^, Seong-Joon Koh^6^, Byung-Soo Kim^7,8,9^* and Kyung-Rok Yu^1,10^*

Correspondence to: [byungskim@snu.ac.kr](mailto:byungskim@snu.ac.kr) and [cellyu@snu.ac.kr](mailto:cellyu@snu.ac.kr)

**This PDF file includes**

Supplementary Materials and Methods

Figs. S1 to S16

Tables S1-2, S4, S7-8

Legends for Tables S3, S5, S6

Uncropped western blots

**Other Supplementary Materials for this manuscript include the following:**

Tables S3 and S5-6**Supplementary Materials and Methods**

**Engineering and Hypoxic Priming of WJ-MSCs**

Human Wharton’s jelly-derived mesenchymal stem cells (WJ-MSCs) were isolated and cultured as previously described.^1^ For gene modification, cells were transduced with a pMX-BMI1 retroviral vector using Lipofectamine 2000 (Thermo Fisher Scientific, Waltham, MA, USA), followed by culture under hypoxic conditions (1% O₂) for 48 h. Overexpression of BMI1 and suppression of p16^INK4a^ were confirmed via qRT-PCR and Western blot. Cell proliferation was evaluated by cumulative population doubling level (cPDL).

**EV Isolation and Characterization**

EVs were collected from conditioned media of engineered WJ-MSCs grown in EV-depleted medium.^2^ Isolation involved serial centrifugation (300 ×g, 2,500 ×g, 10,000 ×g) followed by ultracentrifugation (100,000 ×g). The EV pellet was washed, filtered (0.2 µm), and resuspended in PBS. Size distribution and concentration were measured using nanoparticle tracking analysis (NTA), morphology was assessed by transmission electron microscopy (TEM), and zeta potential was analyzed. EV identity was validated by Western blot detection of CD63, CD81, and absence of calnexin.

**Confocal Imaging of PD-L1 Localization and EV-Cell Interaction**

WJ-MSCs expressing GFP-tagged PD-L1 were stained with WGA-350 (Thermo Fisher Scientific) for 15 min to visualize the cell membrane. Membrane localization of PD-L1 was assessed using confocal microscopy (LSM880; Zeiss, Oberkochen, Germany). For EV–cell interaction, HEK-293T-PD-1-OFP cells were incubated with GFP-labeled PD-L1-enriched EVs for 30 min, and EV binding was evaluated by confocal imaging.

**miRNA Sequencing of EVs**

Total RNA was extracted and libraries were prepared using the SMARTer smRNA-Seq Kit (Takara Bio, Shiga, Japan) and sequenced on an Illumina HiSeq 2500 platform (Illumina, San Diego, CA, USA) (single-end 50 bp). Adapter trimming was performed using Cutadapt (v2.8), and clean reads were aligned to human miRNA references from miRBase v22.1 using miRDeep2.

**Bulk RNA Sequencing of BMI1/Hypoxia-Primed WJ-MSCs**

Total RNA was isolated from BMI1/hypoxia-primed WJ-MSCs using TRIzol reagent (Invitrogen, Carlsbad, CA, USA). mRNA libraries were prepared using the TruSeq Stranded mRNA Library Preparation Kit (Illumina, San Diego, CA, USA) and sequenced on the Illumina HiSeq 2500 (2 × 150 bp). Clean reads were aligned to the human reference genome (GRCh38) using HISAT2 (v2.2.1), and gene expression levels were quantified using featureCounts (v2.0.1).

**Differential Gene Expression and miRNA-mRNA Integration**.

Differentially expressed genes from bulk RNA-seq were identified using EdgeR (log2FC ≥ 0.58, FDR < 0.05), and differentially expressed miRNA from EVs were selected using the same fold-change threshold with p-value < 0.05. Hierarchical clustering and heatmaps were generated in R(v3.6.3) using the ggplot2 package.

For integrative analysis, downregulated mRNAs from BMI1/hypoxia-primed WJ-MSCs were matched with EV-enriched miRNAs. Gene ontology (GO) analysis was performed using Enrichr based on the intersected miRNA–mRNA pairs. miR-27a-3p was selected and target genes were predicted using MIENTURNET, which integrates multiple prediction tools including TargetScan and miRTarBase.

**Transfection of miRNA Mimics and Inhibitors**

Synthetic hsa-miR-27a-3p mimics (30 nM) and negative control (GenePharma, Shanghai, China) were transfected into WJ-MSCs using Lipofectamine RNAiMAX (Invitrogen) according to the manufacturer’s protocol. Mimic overexpression was verified by qRT-PCR for mature miR-27a-3p. For immune cell assays, human PBMCs or human CD4⁺ T cells were transfected with 30 nM miR-27a-3p antisense inhibitors or inhibitor control (GenePharma) using Lipofectamine RNAiMAX in antibiotic-free medium. Downstream assays were performed 48 h post-transfection. Oligonucleotide sequences were: miR-27a-3p inhibitor, 5′-GCGGAACUUAGCCACUGUGAA-3′; negative-control inhibitor, 5′-CAGUACUUUUGUGUAGUACAA-3′. Inhibition efficacy was confirmed by qRT-PCR reduction of mature miR-27a-3p levels.

**Dual-Luciferase Reporter Assay**

PHB1 3′UTR constructs (wild-type and mutant) were cloned into pmirGLO according to manufacturer’s protocol (Promega, Madison, WI, USA). HEK293T cells were co-transfected with the reporter plasmids and either miR-27a-3p mimic or negative control mimic (NC-mimic) using lipofectamine. Luciferase activity was quantified 24 h post-transfection using the Dual-Luciferase Reporter Assay System (Promega).

**RNA Isolation and qRT-PCR**

Total RNA was extracted using TRIzol reagent (Invitrogen). For mRNA analysis, cDNA was synthesized using SuperScript™ III Reverse Transcriptase (Invitrogen). For miRNA analysis, reverse transcription was performed using specific stem-loop RT primers designed for each small non-coding RNA (sncRNA). Quantitative PCR was carried out using miRNA-specific forward primers and a universal reverse primer complementary to the stem-loop sequence. Gene expression was quantified by qRT-PCR, normalized to GAPDH (for mRNA) or U6 (for miRNA), and calculated using the 2^−ΔΔCt^ method.

**Immunoblotting and In vitro binding assay**

Lysates from cells or EVs were analyzed by SDS-PAGE and immunoblotting using antibodies against various suppliers including PD-L1 (Thermo Fisher Scientific), GFP, GRB2, CD81, SHP2, COX2, GAPDH (Santa Cruz Biotechnology, Dallas, TX, USA), PHB1 (GeneTex, Irvine, CA, USA), CD63 (System Biosciences, Palo Alto, CA, USA), Calnexin, p-ZAP70, ZAP70, p-AKT, AKT, BMI1 (Cell Signaling Technology, Danvers, MA, USA), p16^INK4a^ ,TGF-β (Abcam, Cambridge, UK), IDO (Millipore, Burlington, MA, USA). Detection was performed using HRP-conjugated secondary antibodies and ECL substrate (Thermo Fisher Scientific). Immunoblots were analyzed in ImageJ. For each lane, target band intensity was background-subtracted and normalized to the lane’s loading control (GAPDH/β-actin/ZAP70/AKT for cells; CD63/TSG101 for EVs), then scaled to the reference condition (1.0). Technical replicates were averaged within each biological replicate.

**Public dataset analysis**

Public PBMC transcriptomic data (GSE3365; Burczynski et al., 2006, PMID: 16436634) were analyzed using normalized expression values from the Affymetrix Human Genome U133A Array (GPL96). Group differences were tested by one-way ANOVA with Dunnett’s post hoc correction after verifying normality.

**Transepithelial-Transendothelial Electrical Resistance (TEER) and FITC dextran permeability assays**

Caco-2 monolayers were seeded onto collagen IV-coated Transwell inserts (0.4 µm pore, 4 × 10^4^ cells/insert) and cultured until stable baseline TEER (< 10% variation). EVs (100 µg/mL) were added apically for 48 h. For inflammatory stress, TNF-α + IFN-γ (10 ng/mL each, apical + basolateral) was applied for 48 h. TEER was measured at 0, 6, 24, 48 h using an EVOM3 epithelial volt-ohm meter (World Precision Instruments LLC., Sarasota, FL, USA). Resistance was corrected by blank subtraction. TEER(Ω⋅cm2)=(R sample​−R blank​)×A. After 48 h, FITC-dextran (4 kDa, 1 mg/mL) was added apically and basolateral fluorescence (Ex/Em 485/520 nm) were recorded after 2 h.

**Immunofluorescence staining**

Cells on chamber slides were fixed with 4% PFA (15 min, RT), permeabilized (0.2% Triton X-100, 5 min), and blocked (5% BSA, 30 min). Samples were incubated overnight at 4 °C with primary antibodies against ZO-1 (AF5145, Affinity Biosciences, Cincinnati, OH) and occludin (E6B4R, Cell Signaling Technology, Danvers, MA), followed by Alexa Fluor–conjugated secondary antibodies (1:500, 1 h, dark). Nuclei were counterstained with DAPI and mounted with DAKO medium (Glostrup, Denmark). Images were acquired using a Leica TCS SP8 X confocal microscope (Leica Microsystems, Wetzlar, Germany).

**Apoptosis assay**

Adherent and floating cells were collected, stained with Annexin V-FITC and 7-AAD in binding buffer (2.5 mM Ca²⁺, 15 min, RT, dark), and analyzed immediately by flow cytometry.

**Fluo-4 Calcium Flux Assay.**

Human CD4⁺ T cells were pre-activated with anti-CD3/CD28 for 16 h, washed, and incubated with EVs for 1 h at 37 °C. Intracellular Ca²⁺ flux was measured using the Fluo-4 Direct™ Calcium Assay Kit (Thermo Fisher) on a VICTOR Nivo plate reader. Fluorescence (Ex 494/Em 516 nm) was recorded every 2 s for 7 min. Signals were normalized to baseline (ΔF/F₀). Ionomycin was used as a positive control. Increased fluorescence indicates Ca²⁺ influx.

**EV Biodistribution Imaging**

EVs were fluorescently labeled with Cy5.5 NHS ester dye (10 mM; Abcam, Cambridge, MA, USA) and intravenously injected into humanized colitis mice. At 24 h post-injection, organs were harvested, and whole-body fluorescence imaging was performed using the IVIS Spectrum (PerkinElmer, Waltham, MA, USA). Signal intensities were quantified with Living Image software.

**In Vivo CD4⁺ T Cell Analysis and Cytokine ELISA in Humanized Mice**

Human CD4⁺ T cells were isolated from peripheral blood, spleen, and colon tissues of humanized mice, as previously described. ^2^ Tissues were dissociated, passed through a 70 μm cell strainer, and red blood cells were lysed using RBC lysis buffer (eBioscience). Human CD4⁺ T cells were purified by MACS (Miltenyi Biotec) according to the manufacturer’s protocol. Cells were stained with antibodies against human CD45, CD4, IFN-γ, IL-17A, CD25, and FOXP3 (BD Biosciences). To exclude murine cells, anti-mouse CD45 antibody was included. Flow cytometry was used for T cell subset analysis. Serum cytokines (IL-10, IFN-γ, TNF-α) were quantified using human-specific ELISA kits (R&D Systems, Minneapolis, MN, USA) according to the manufacturer’s instructions.

**Histology and Immunohistochemistry**

Colon tissues were formalin-fixed, paraffin-embedded, and sectioned at 5 µm. Sections were stained with hematoxylin and eosin (H&E) or immunostained with anti-human CD3 (DAKO, Glostrup, Denmark) followed by DAB substrate (Vector Laboratories, Burlingame, CA, USA). Images were quantified using ImageScope software (Leica Biosystems, Buffalo Grove, IL, USA).

**scRNA-seq Data Processing and Analysis**

Raw sequencing data were processed using Cell Ranger v6.1.1 (10x Genomics) pipeline with the GRCh38(Ensemble v113) human reference genome. Empty droplets were excluded using the DropletUtils R package (v2.0),^3^ low-quality cells (UMI < 600, gene count < 100, or >30% mitochondrial content) were filtered out. After quality control, samples merged for downstream analysis. Normalization was performed using log₂-transformation with a pseudocount of 1 following size factors estimation by the scran package.^4^ Highly variable genes (HVGs) were selected using the modelGeneVar, and Principal component analysis (PCA) was conducted using Seurat v4.3.0. ^5^ Batch effects were corrected using Harmony package (v0.1.1) on the top 10 principal components.^6^

Cell clustering was performed using the Louvain algorithm with multiple resolution settings (0.2–0.5), and dimensionality reduction and visualization were achieved via Uniform Manifold Approximation and Projection (UMAP). Clusters were manually annotated based on canonical marker genes and reference literature**.** Marker genes were identified with Seurat’s FindAllMarkers and FindMarkers functions (Wilcoxon rank-sum test; |log₂FC| and FDR thresholds applied). Rare non-target clusters (“B cell-like” and “ambiguous,” each < 2% of total cells) were retained for visualization but excluded from quantitative analyses. To assign donor identity and remove doublets, souporcell v2.4 was used with donor genotype references, and only high-confidence singlets were retained for analysis. ^7^

Marker gene expression was visualized using DotPlot and FeaturePlot functions in Seurat, and marker selection was curated to represent well-established CD4⁺ T cell subset features, including naïve/memory (IL7R), cytotoxic (GZMB), regulatory (FOXP3, IL2RA), and proliferative (MCM6, CDK1) populations.

For quantitative comparisons between treatment groups, the proportion of each annotated cell type was calculated and statistically compared using unpaired two-tailed Student’s t-test. Pseudobulk gene expression matrices were generated per sample and cell type using the glmGamPoi package, and differential gene expression analysis was performed using DESeq2 (v1.40.2). The model design included donor sample as a covariate (~ sample + condition), and differentially expressed genes were defined by an adjusted p-value < 0.05 and |log₂FC| > 1.

For functional enrichment analysis, upregulated and downregulated gene sets (log₂FC > 0.58) were analyzed using Gene Ontology (GO) Biological Process and curated pathway databases (KEGG and Reactome) through EnrichR. Enrichment was calculated using default parameters with FDR correction.

Because the input consisted of prospectively purified human CD4⁺ T cells, the resulting scRNA-seq dataset represents T cell intrinsic transcriptional programs. Non-T cell clusters were minimal and are shown only for completeness.

**Supplementary Figures**

**Figure S1.**


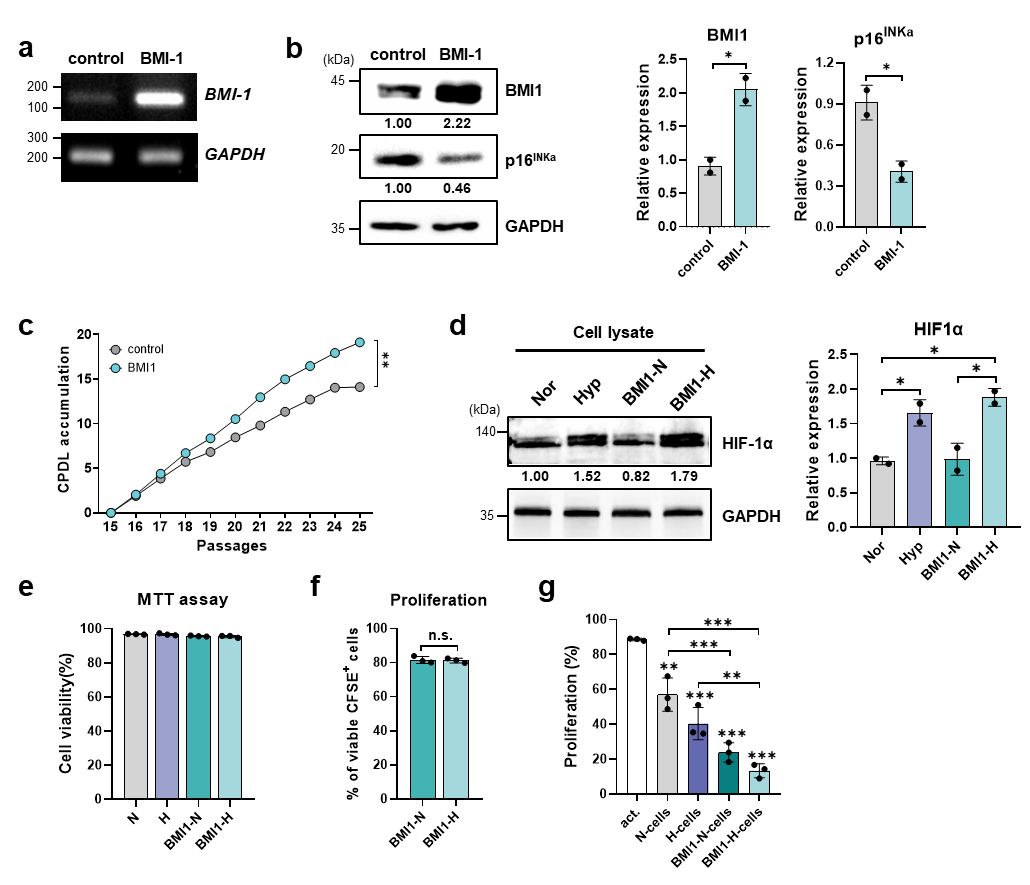


**Supplementary Figure 1.** Characterization of BMI1-overexpressing and hypoxia-conditioned WJ-MSCs. **a** RT-PCR analysis of BMI1 expression in control and BMI1-overexpressing WJ-MSCs. **b** Western blot analysis of BMI1 and p16INK4a protein expression in control and BMI1-overexpressing WJ-MSCs. GAPDH was used as a loading control. **c** Cumulative population doubling levels (CPDL) measured over serial passages. **d** Western blot analysis of HIF-1α expression in WJ-MSCs cultured under normoxia (Nor), hypoxia (Hyp), BMI1-normoxia (BMI1-N), and BMI1-hypoxia (BMI1-H) conditions. Band intensities were quantified in ImageJ and normalized to GAPDH, relative values and corresponding bar graphs (mean ± SD, n = 2) are shown. **e** MTT assay assessing cell viability under the indicated conditions. **f** Flow cytometric quantification of proliferating (7-AAD⁻CFSE⁺) WJ-MSCs cultured under BMI1-N and BMI1-H conditions. **g** Flow cytometric analysis of CFSE-labeled activated T cell proliferation following co-culture with WJ-MSCs under Nor, Hyp, BMI1-N, and BMI1-H conditions. Data represent the mean ± SD from n = 3 independent biological replicates. Technical triplicates were averaged prior to statistical analysis. Statistical significance was determined using paired Student’s t-test or one-way ANOVA with Tukey’s multiple comparisons test. * *p* < 0.05, ** *p* < 0.01, *** *p* < 0.001, n.s.: not significant.

Figure S2.


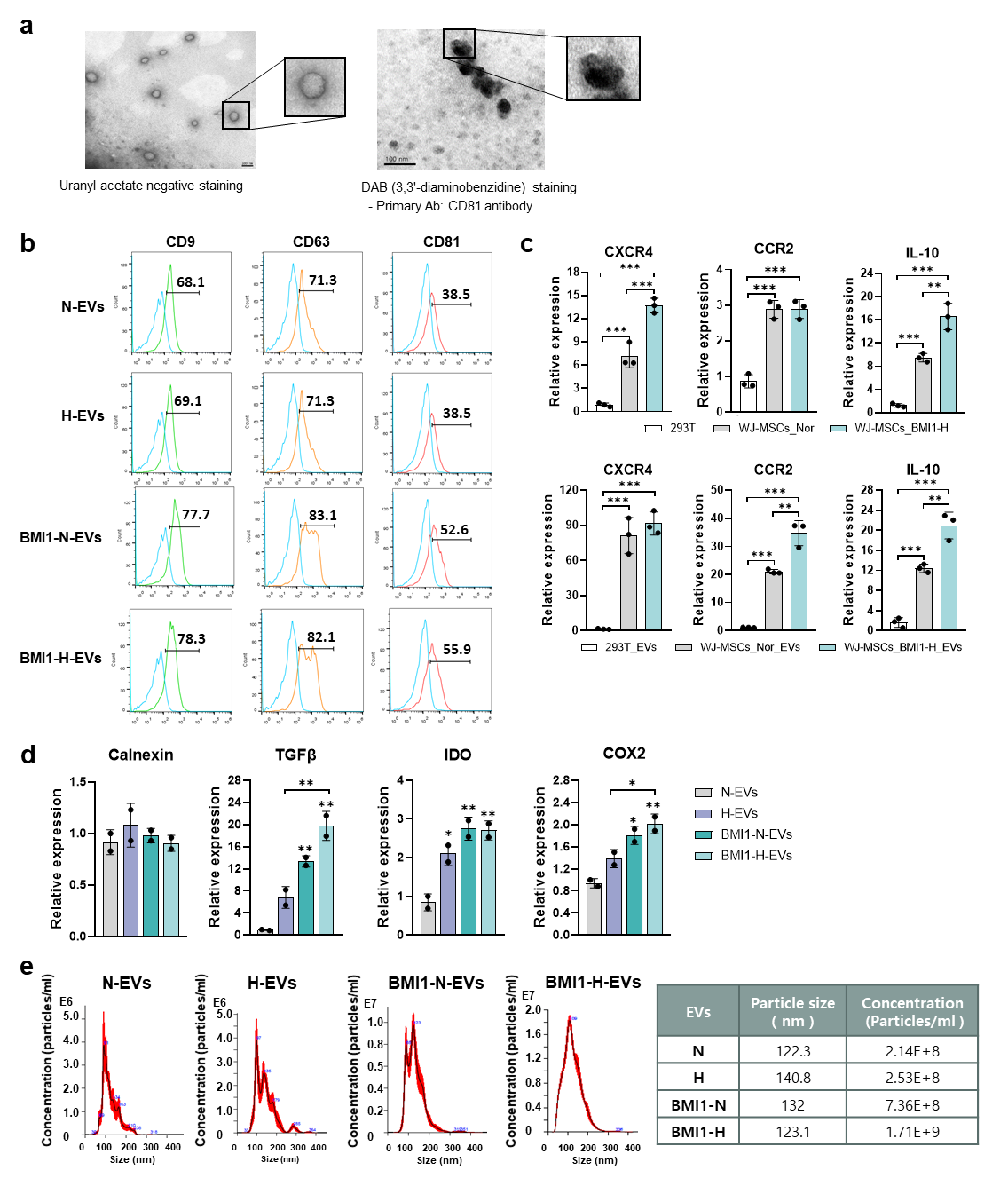


**Supplementary Figure 2.** Characterization of EVs derived from BMI1/hypoxia primed WJ-MSCs. **a** TEM images of EVs stained with uranyl acetate (left) and immunogold labeling of CD81 visualized by DAB staining (right). **b** Flow cytometric analysis of EV surface marker expression (CD9, CD63, and CD81) across different treatment groups. **c, d** Quantitative densitometry of immunoblots shown in Fig. 1b and Fig. 1c, respectively. Band intensities were quantified in ImageJ and normalized to the corresponding loading controls (cells: GAPDH; EVs: CD63). Bar graphs summarize normalized protein expression (mean ± SD, n = 2-3) across biological replicates. **e** NTA showing particle size distribution and concentration of EVs derived from indicated conditions. Representative TEM, flow cytometry, and NTA data are shown from three independent biological replicates. No formal statistical analysis applied. Statistical significance was determined using one-way ANOVA with Tukey’s multiple comparisons test. * *p* < 0.05, ** *p* < 0.01.

Figure S3.


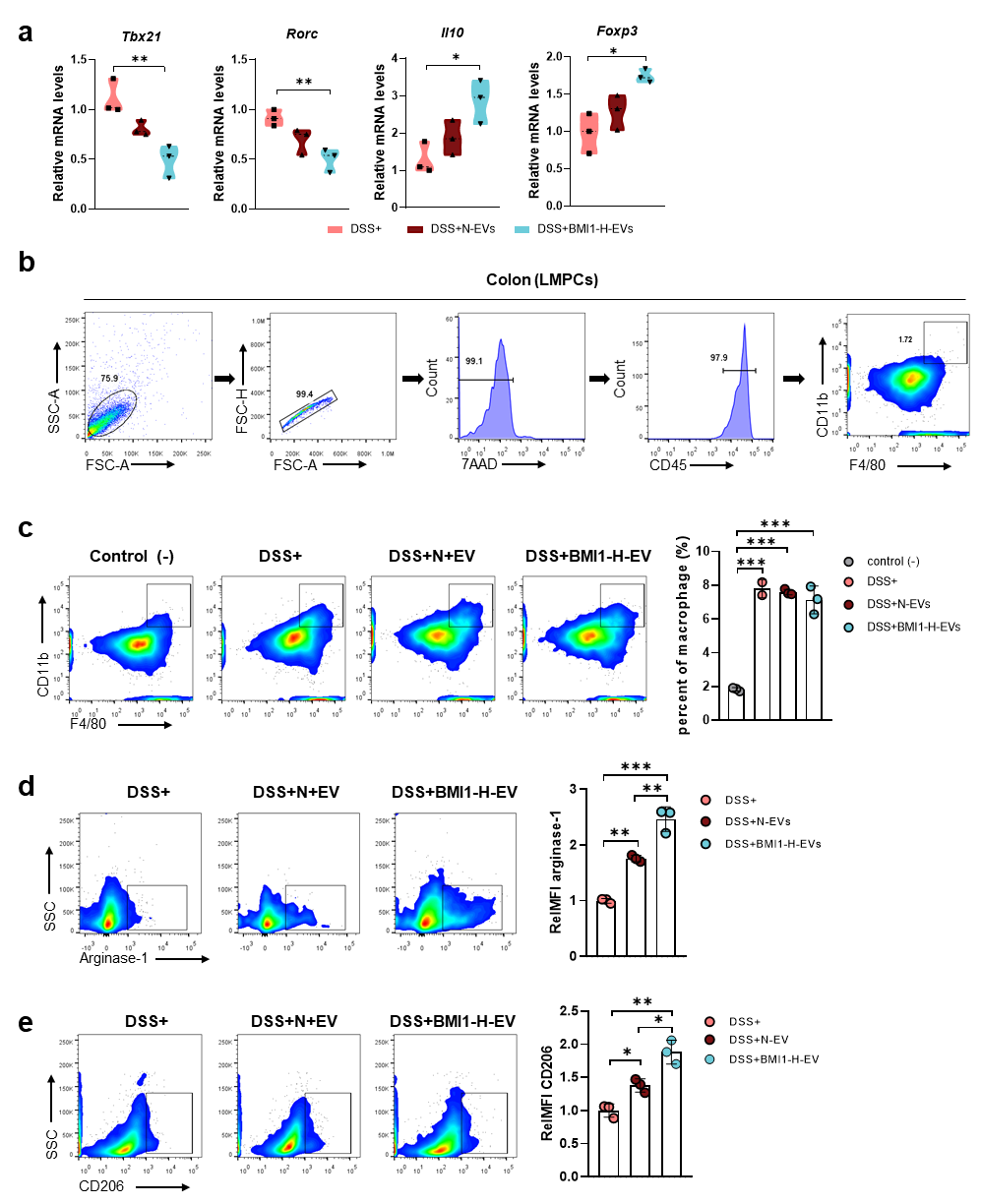


**Supplementary Figure 3.** Effects of BMI1/hypoxia-primed EVs on T cell and macrophage responses in DSS-induced colitis. **a** qRT-PCR analysis of *Tbx21* (Th1), *Rorc* (Th17), *Il10*, and *Foxo3* (Treg) mRNA expression in colonic tissues from DSS-treated mice following treatment with normoxia-derived EVs (N-EVs) or BMI1/hypoxia-primed EVs (BMI1-H-EVs). **b** Gating strategy for the identification of colonic lamina propria macrophages by flow cytometry. Cells of the monocyte-macrophage lineage were defined within the live (7-AAD^-^) single CD45⁺CD11b⁺F4/80⁺ population. **c** Representative flow cytometry plots and quantification showing the frequency of macrophages among lamina propria cells in control (untreated), DSS, DSS+N-EV, and DSS+BMI1-H-EV groups. **d, e** Representative plots and quantification of M2-like macrophage markers Arg1 (**d**) and CD206 (**e**) in colonic macrophages from DSS-challenged mice treated with N-EVs or BMI1-H-EVs, along with relative mean fluorescence intensity (MFI) normalized to the DSS group. Data are presented as mean ± standard deviation (SD) from n = 3 mice per group. Statistical significance was determined using one-way ANOVA followed by Tukey’s multiple comparisons test. * *p* < 0.05, ** *p* < 0.01, *** *p* < 0.001

Figure S4.


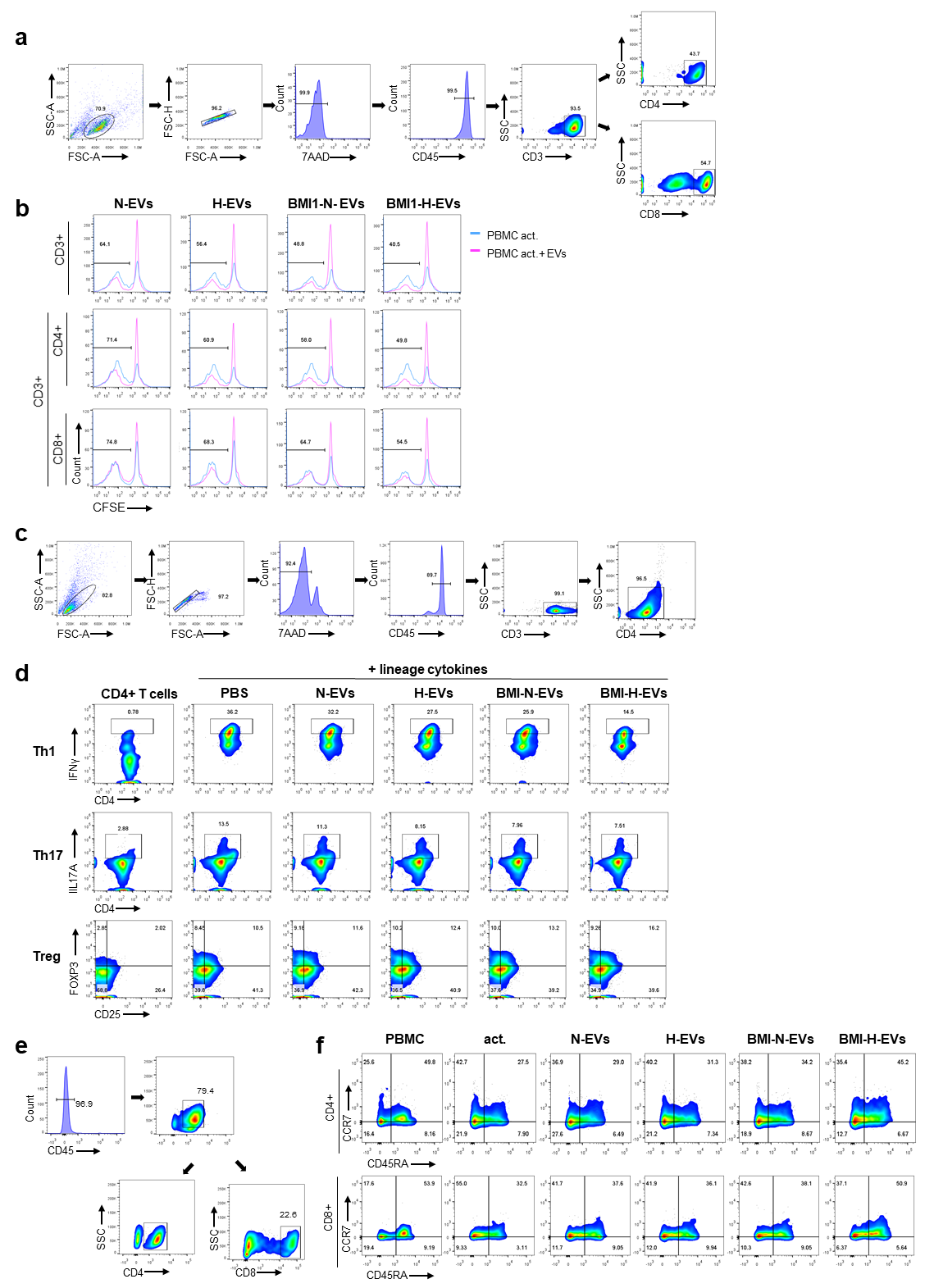


**Supplementary Figure 4.** Immunomodulatory effects of BMI1/hypoxia-primed EVs on human CD4⁺ T cell activation and lineage polarization. **a** Gating strategy for the PBMC proliferation assay: FSC/SSC pre-gate → singlets (FSC-A vs FSC-H) → live cells (7-AAD^-^) → CD45⁺ leukocytes → CD3⁺ T cells→CD4⁺ and CD8⁺ subsets. CFSE dilution was quantified within each gate population. **b** Representative CFSE histograms for total CD3⁺, CD4⁺, and CD8⁺ T cells after anti-CD3/28 stimulation in the presence of the indicated EV preparations (N-EVs, H-EVs, BMI1-N-EVs, BMI1-H-EVs). Percentage values indicate the fraction of proliferated (CFSE low) cells within each subset. **c** Gating strategy for analysis of activated T cell subsets using sorted CD4⁺ T cells: FSC/SSC → singlets → live (7-AAD^-^) → CD45⁺ → CD3⁺ → CD4⁺. Human CD4⁺ T cell isolated from PBMCs were stimulated with anti-CD3/28 under lineage-polarizing conditions and subsequently processed for intracellular cytokine staining (PMA/ionomycin, brefeldin A) to identify CD4⁺CD25⁺FOXP3⁺ Treg, CD4⁺IFNγ⁺ Th1 cells, and CD4⁺IL-17A⁺Th17 cells. **d** Representative intracellular cytokine staining plots showing Th1 (IFNγ⁺), Th17 (IL-17A⁺), and Treg (FOXP3⁺) populations among CD4⁺ T cells following treatment with PBS, N-EVs, H-EVs, BMI1-N-EVs, or BMI1-H-EVs under lineage cytokines treatment conditions. **e** Gating for memory phenotype analysis of human CD4⁺ and CD8⁺ T cells based on CD45RA and CCR7 expression: live CD45⁺CD3⁺ T cells → CD4⁺ or CD8⁺ → memory subsets defined by CD45RA and CCR7 expression. **f** Representative density plots of memory T cell phenotypes in CD4⁺ and CD8⁺ T cells, including naïve (TN, CD45RA^+^CCR7^+^), central memory (TCM, CD45RA^-^CCR7^+^)**,** effector memory (TEM, CD45RA^-^CCR7^-^)**,** and terminal effector (TEFF, CD45RA^+^CCR7^-^) populations. Positivity thresholds were defined using fluorescence minus one (FMO) controls. Plots are representative of n = 3 independent donors. Quantitative summaries are presented in the main figures.

Figure S5.


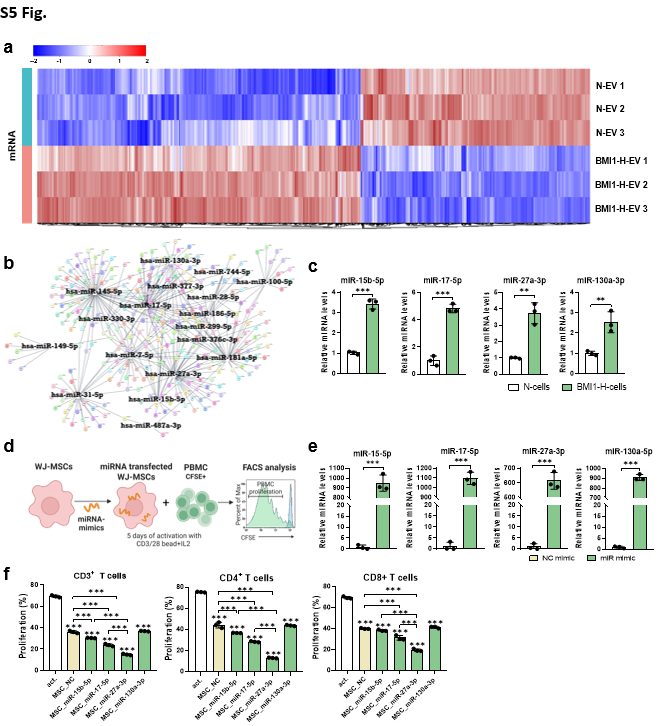


**Supplementary Figure 5.** Differential expression and immunomodulatory function of miRNAs in BMI1/hypoxia-primed WJ-MSCs and their EVs. **a** Heatmap of differentially expressed genes (DEGs) in BMI1/hypoxia-primed WJ-MSCs (BMI1-H) compared to normoxia-cultured WJ-MSCs (N-cells). Each column represents an independent biological replicate (n = 3 per group). **b** Network analysis of 19 differentially expressed miRNAs (DE-miRNAs) and their predicted target genes constructed using miRNet. **c** qRT-PCR analysis of selected miRNA (miR-27a-3p, miR-17-5p, miR-130a-3p, and miR-15b-5p) in N-cells and BMI1-H cells. **d** Schematic overview of the experimental workflow for evaluating the immunomodulatory function of miRNA mimic-transfected WJ-MSCs. **e** qRT-PCR validation of miRNA overexpression in WJ-MSCs following transfection with individual synthetic miRNA mimics. **f** CFSE-based T cell proliferation assay following 5-day co-cultured with miRNA mimic-transfected WJ-MSCs. Proliferation was assessed by flow cytometry. Data represent n = 3 independent biological replicates. Technical triplicates for qRT-PCR and T cell assays were averaged before statistical analysis. Statistical comparisons were performed using paired Student’s t-test or one-way ANOVA with Tukey’s post hoc test. * *p* < 0.05, ** *p* < 0.01, *** *p* < 0.001.

Figure S6.


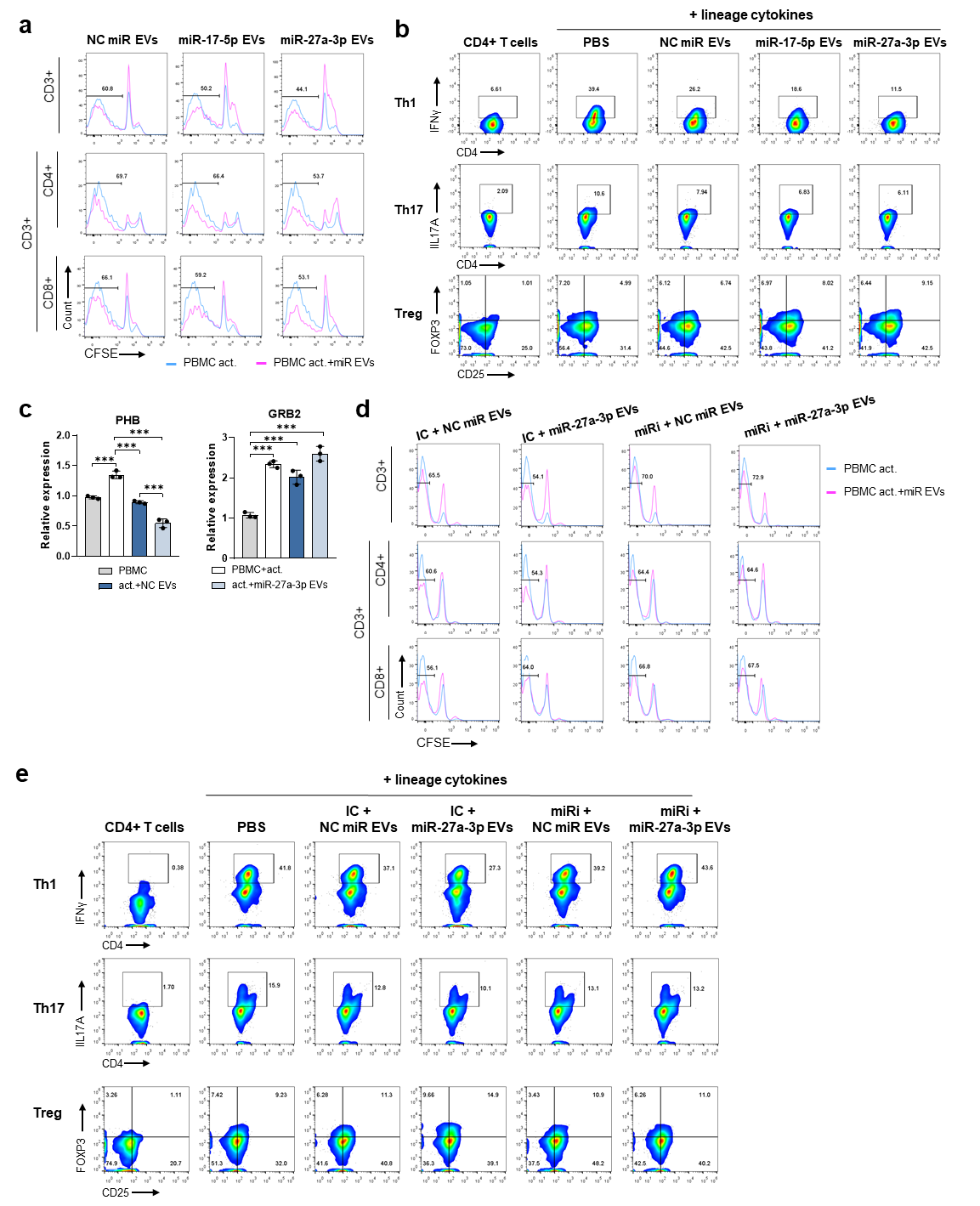


**Supplementary Figure 6.** Functional validation of miR-27a-3p-enriched EVs in modulating human T cell proliferation and lineage polarization. **a** CFSE-based proliferation assay of activated PBMCs co-cultured with EVs derived from WJ-MSCs transfected with negative control (NC) miRNA, miR-17-5p, or miR-27a-3p mimics. CFSE dilution was quantified in total CD3⁺, CD4⁺, and CD8⁺ T cell populations. **b** Representative intracellular cytokine staining plots showing Th1 (IFNγ⁺), Th17 (IL-17A⁺), and Treg (CD25⁺FOXP3⁺) populations among CD4⁺ T cells following treatment with PBS, NC-miR EVs, miR-17-5p EVs, or miR-27a-3p EVs under lineage cytokines treatment conditions. **c** Quantitative densitometry of immunoblots shown in Fig. 3d. Band intensities were quantified in ImageJ and normalized to GAPDH. Bar graphs summarize normalized protein expression (mean ± SD, n = 3) across biological replicates. Statistical comparisons were performed using one-way ANOVA with Tukey’s post hoc test. * *p* < 0.05, ** *p* < 0.01. **d**. CFSE-based proliferation analysis of activated PBMCs co-cultured with inhibitor control (IC) or miR-27a-3p inhibitor in the presence or absence of miR-27a-3p EVs. The percentage of proliferated (CFSE low) cells is indicated for each T cell subset. **e** Representative intracellular cytokine staining plots of CD4⁺ T cells showing Th1 (IFNγ⁺), Th17 (IL-17A⁺), and Treg (CD25⁺FOXP3⁺) populations following co-culture with inhibitor control (IC) or miR-27a-3p inhibitor in the presence or absence of miR-27a-3p EVs. Positivity thresholds were defined using FMO controls. Plots are representative of n = 3 independent donors. Quantitative summaries are presented in the main figures.

Figure S7.


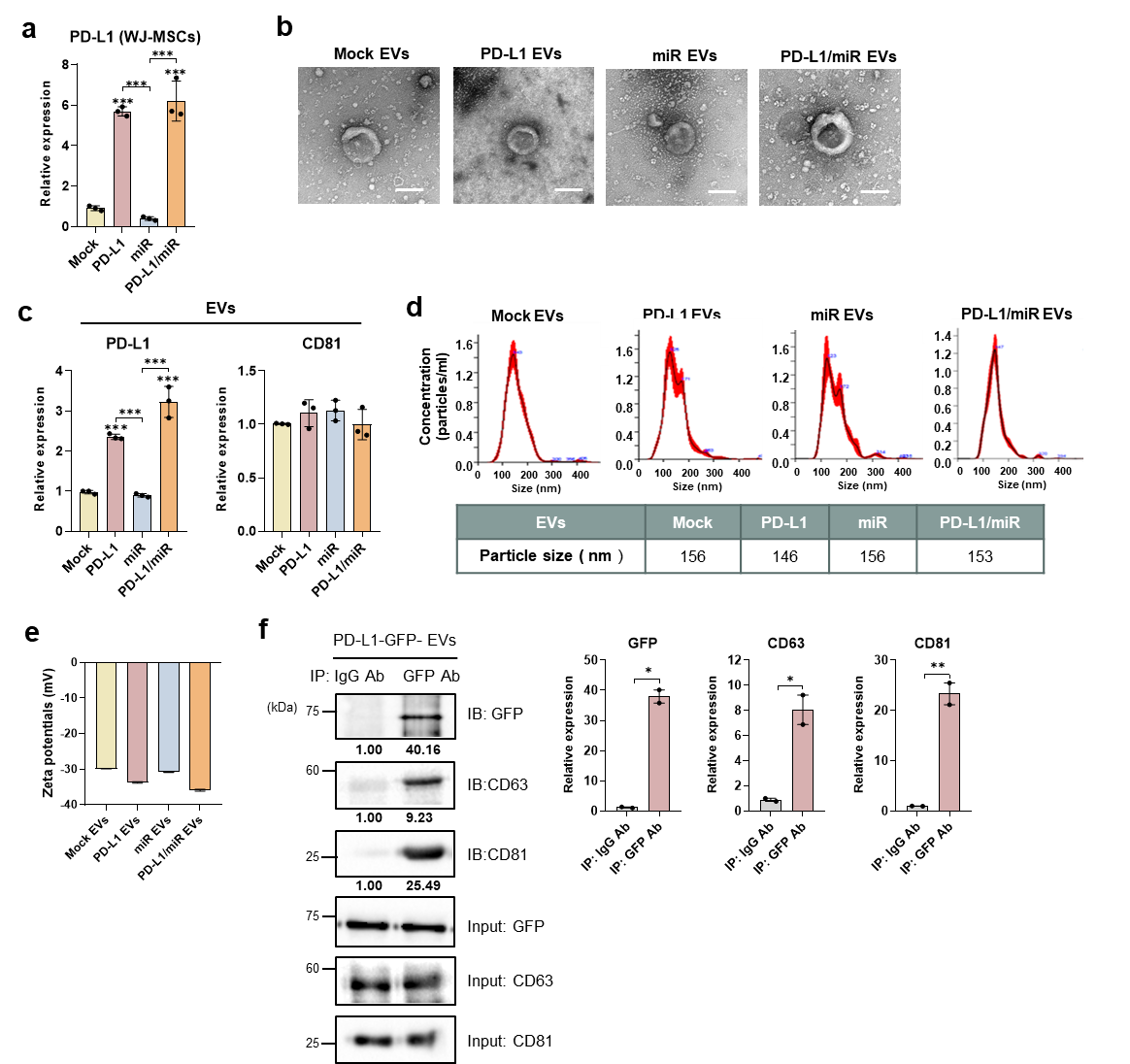


**Supplementary Figure 7.** Characterization of engineered EVs by TEM, NTA, zeta potential, and immunoblot analysis. **a** Quantitative densitometry of immunoblots shown in Fig. 4c. Band intensities were quantified in ImageJ and normalized to β-actin. Bar graphs summarize normalized protein expression (mean ± SD, n = 3) across biological replicates. **b** Representative TEM images of engineered EV derived from different groups. Scale bars: 100 nm. **c** Quantitative densitometry of immunoblots shown in Fig. 4f. Band intensities were quantified in ImageJ and normalized to CD63. Bar graphs summarize normalized protein expression (mean ± SD, n = 3) across biological replicates. **d** NTA revealing comparable size distribution profiles among EV groups. **e** Zeta potential measurements for EV surface charge characterization. **f** Immunoprecipitation (IP) of PD-L1-GFP⁺ EVs using GFP antibodies, followed by immunoblotting (IB) for GFP and CD63. Input represents total EV lysates. Band intensities were quantified in ImageJ and normalized to input control (GFP, CD63, CD81) and corresponding bar graphs (mean ± SD, n = 2) are shown. Statistical comparisons were performed using paired Student’s t-test or one-way ANOVA with Tukey’s post hoc test. * p < 0.05, ** p < 0.01. Representative TEM, NTA, and immunoprecipitation results from at least three independent experiments. No statistical analysis was applied.

Figure S8.

**
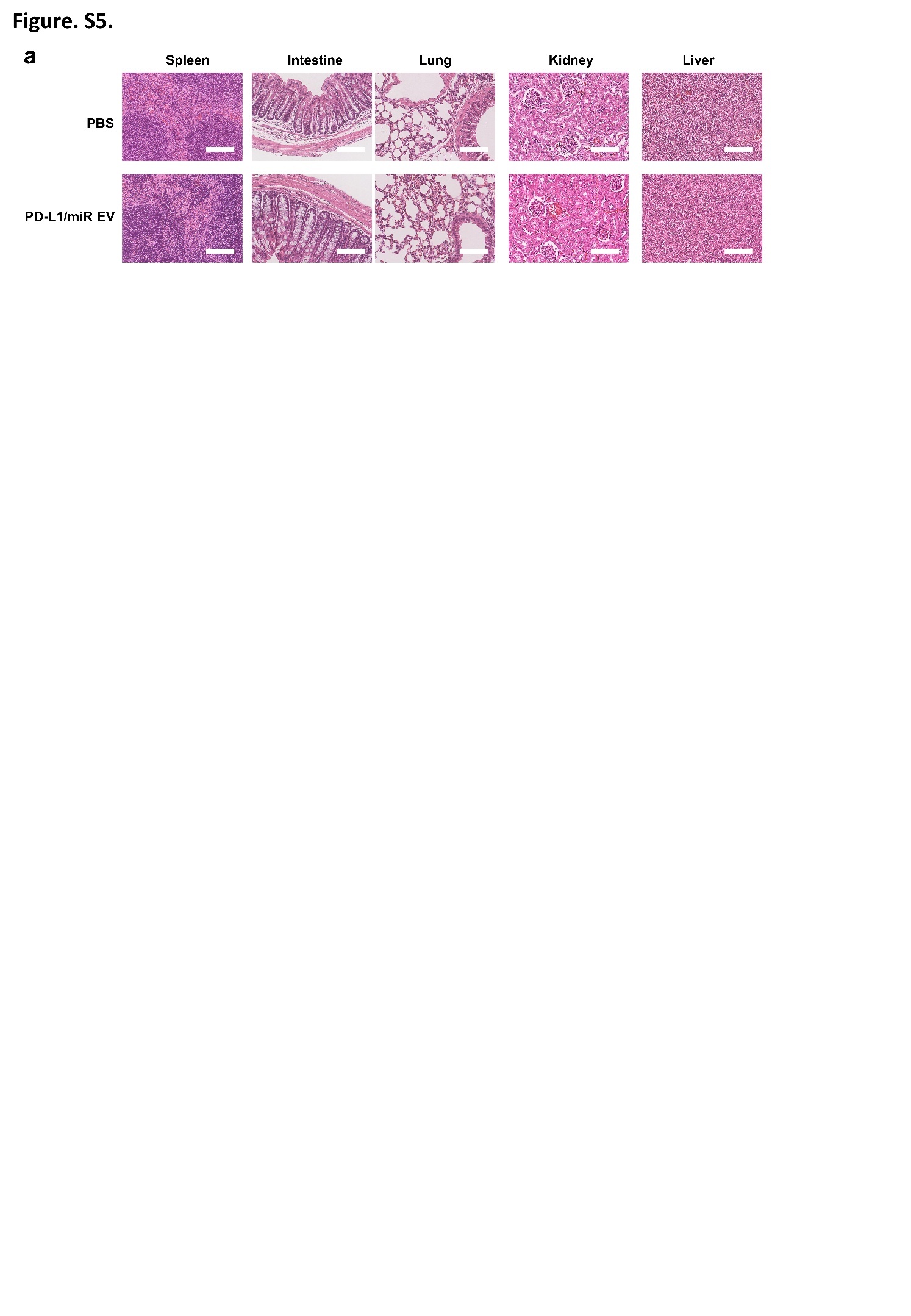
 Supplementary Figure 8.** Histological analysis of major organs following systemic PD-L1/miR-27a-3p EV administration. **a** Representative H&E-stained sections of spleen, colon, lung, kidney, and liver harvested 7 days after i.v. injection of PBS (top row) or PD-L1/miR-27a-3p-enriched EVs (bottom row). Scale bars: 100 μm. Representative histological images from n = 3 mice per group. No quantitative statistics performed.

**Figure S9.**


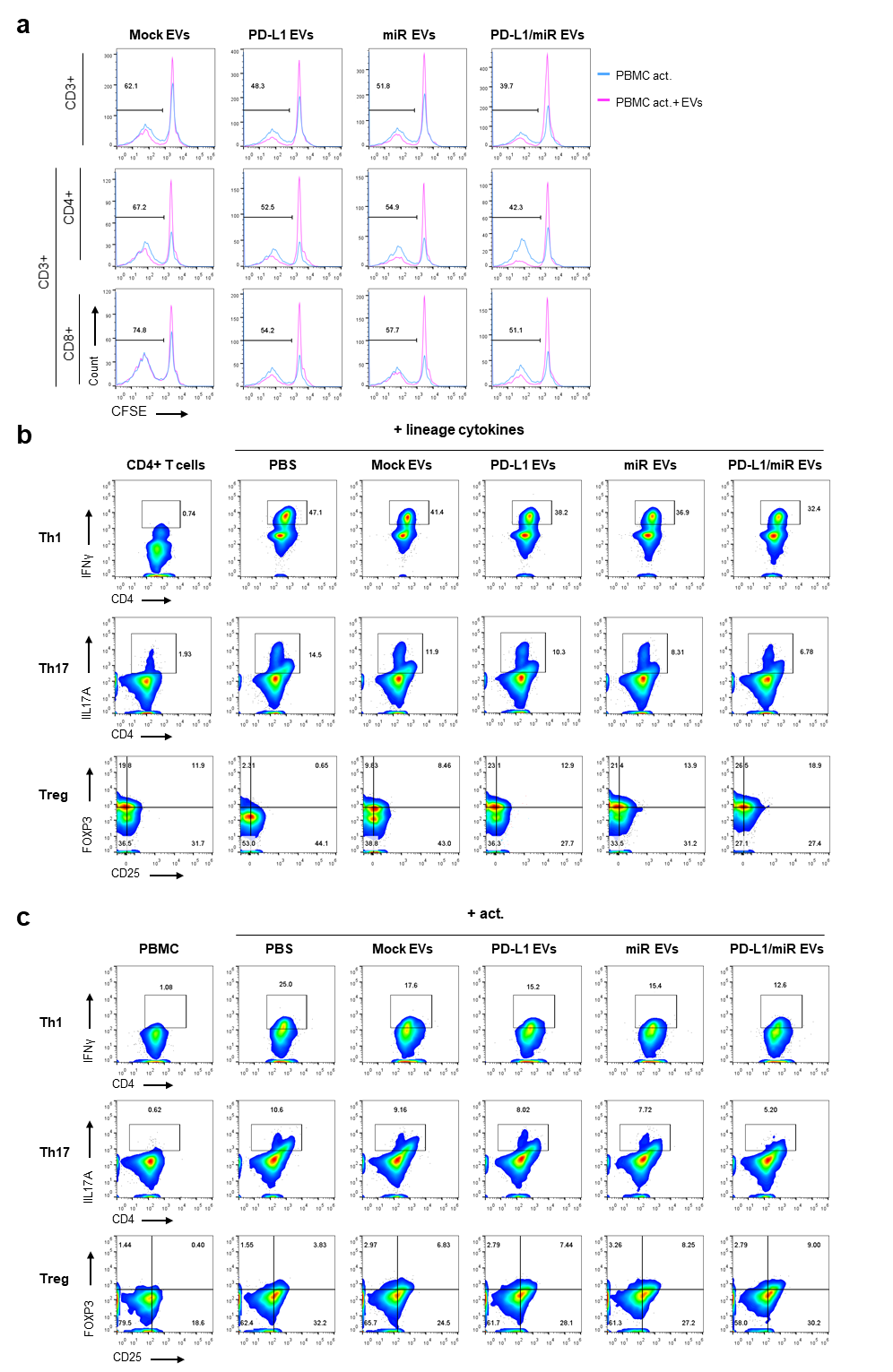


**Supplementary Figure 9.** Functional evaluation of PD-L1/miR-27a-3p-enriched EVs in activated human PBMCs and CD4⁺ T cells**. a** CFSE-based proliferation assay of activated PBMCs co-cultured with EVs derived from mock-, PD-L1-, miR-27a-3p-, or PD-L1/miR-27a-3p engineered WJ-MSCs. CFSE dilution was analyzed in total CD3⁺, CD4⁺, and CD8⁺ T cell populations. **b** Representative intracellular cytokine staining plots showing Th1 (IFNγ⁺), Th17 (IL-17A⁺), and Treg (CD25⁺FOXP3⁺) populations among CD4⁺ T cells following treatment with PBS, control EVs, PD-L1 EVs, miR-27a-3p EVs, or PD-L1/miR-27a-3p EVs under lineage cytokines treatment conditions. **c** Representative intracellular cytokine staining plots showing Th1, Th17, and Treg subsets in activated CD4⁺ T cells co-cultured with PD-L1/miR-27a-3p EVs under PBMC activation conditions. Positivity thresholds were defined using FMO controls. Plots are representative of n = 3 independent donors. Quantitative analyses are presented in the main figures.

Figure S10.


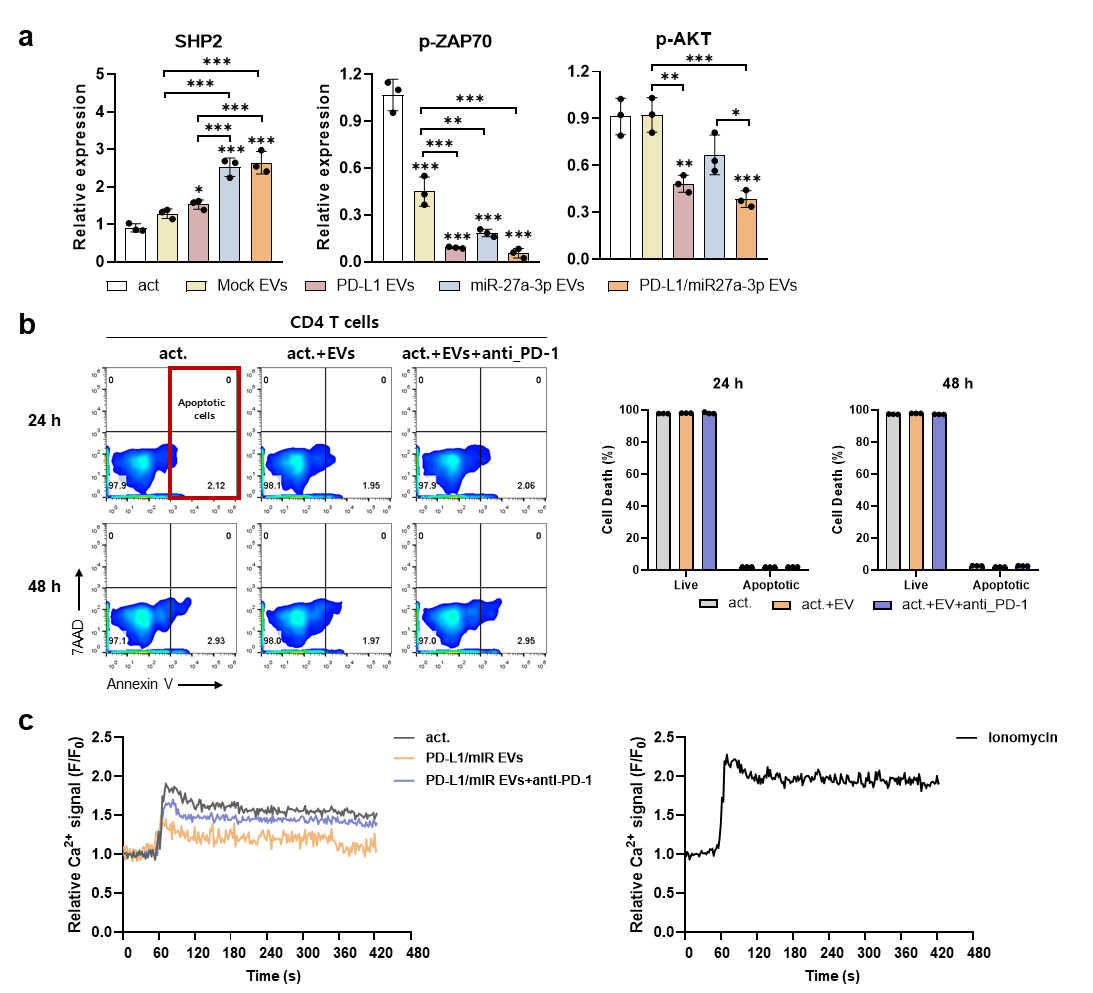


**Supplementary Figure 10.** PD-L1/miR-27a-3p-enriched EVs suppress TCR signaling while preserving T-cell viability. **a** Quantitative densitometry of immunoblots shown in Fig. 4o. Band intensities were quantified in ImageJ and normalized to the corresponding loading controls (β-actin, ZAP70, AKT). Bar graphs summarize normalized protein expression (mean ± SD, n = 3 biological replicates). Statistical comparisons were performed using one-way ANOVA with Tukey’s post hoc test. * p < 0.05, ** p < 0.01, *** p < 0.001. **b** Flow cytometric analysis of Annexin V/7-AAD staining in activated CD4⁺ T cells following treatment with PBS or PD-L1/miR-27a-3p EVs, with or without PD-1 blockade. Representative plots show Annexin V⁺7-AAD⁻/⁺ populations at 24 h, and bar graphs summarize apoptotic cell frequencies at 24 h and 48 h. Statistical analysis was performed using one-way ANOVA with Tukey’s multiple comparisons test; no statistically significant differences were observed among groups. **c** Fluo-4 based calcium flux assay of activated CD4⁺ T cells co-cultured with PD-L1/miR-27a-3p EV, in the presence or absence of PD-1 blockade. Ionomycin stimulation served as a positive control. Data represent n = 3 independent biological replicates. Representative flow cytometry plots and calcium flux traces are shown.

Figure S11.


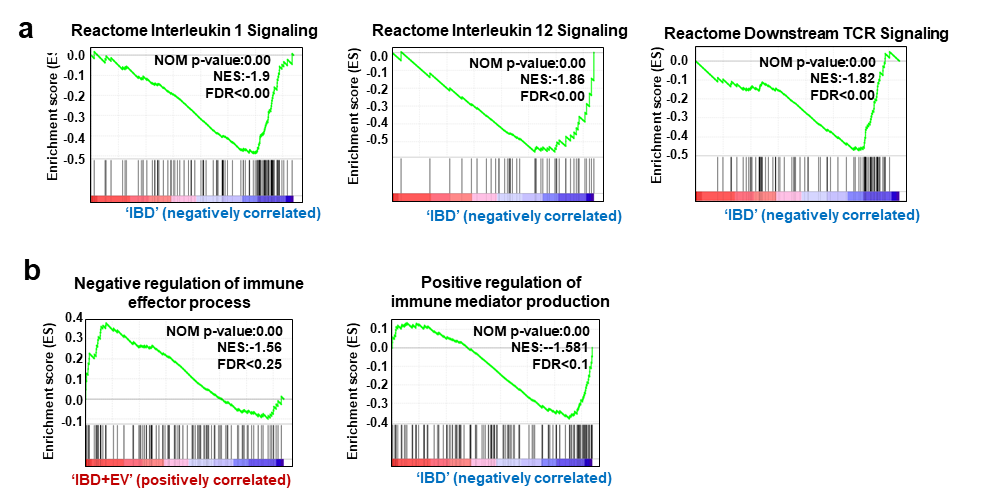


**Supplementary Figure 11.** Transcriptomic features associated with immune effector regulation and T cell exhaustion in CD4⁺ T cells from patients with IBD following treatment with PD-L1/miR-27a-3p-enriched EVs. **a KEGG and reactome pathway enrichment analyses indicating suppression of IL-1, IL-12, interferon, and TCR signaling pathways in PD-L1/miR-27a-3p EV-treated cells. b** **Gene Set Enrichment Analysis (GSEA) plots showing downregulation of immune effector process and immune mediator production gene sets in PD-L1/miR-27a-3p EV-treated CD4⁺ T cells compared with untreated controls. NES, normalized enrichment score; FDR, false discovery rate; NOM p-value, nominal p-value. Transcriptomic data represent n = 3 independent biological replicates. GSEA was performed using default statistical parameters in the Broad Institute software.**

Figure S12


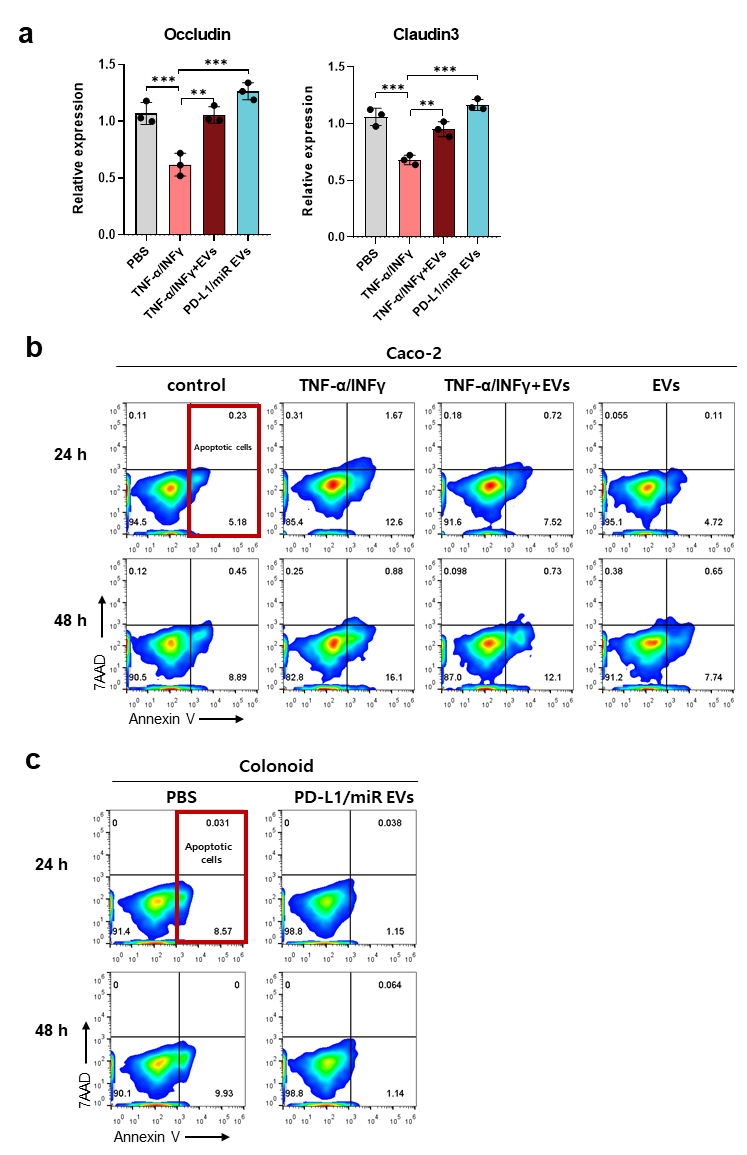


**Supplementary Figure 12.** Analysis of apoptosis in Caco-2 and colonoid-derived epithelial monolayers treated with PD-L1/miR-27a-3p-enriched EVs. **a** Quantitative densitometry of immunoblots shown in Fig. 6c. Band intensities were quantified in ImageJ and normalized to GAPDH. Bar graphs summarize normalized protein expression (mean ± SD, n = 3) across biological replicates. Statistical comparisons were performed using one-way ANOVA with Tukey’s post hoc test. * p < 0.05, ** p < 0.01. **b** Annexin V/7-AAD flow cytometric analysis of Caco-2 monolayers cultured in EV-depleted medium under basal or inflammatory conditions (TNF-α/IFN-γ, 10 ng/mL each) with or without PD-L1/miR-27a-3p-enriched EVs (100 μg/mL) for 24 h and 48 h. Representative plots show Annexin V⁺7-AAD⁻ (early apoptotic) and Annexin V⁺7-AAD⁺ (late apoptotic/necrotic) populations at 24 h and 48 h. **c** Representative Annexin V/7-AAD staining of colonoid-derived monolayers at 24 h and 48 h, showing live and apoptotic cell fractions. Data represent n = 3 independent donors, analyzed in technical triplicates. Quantitative analyses are presented in the main figures.

Figure S13.


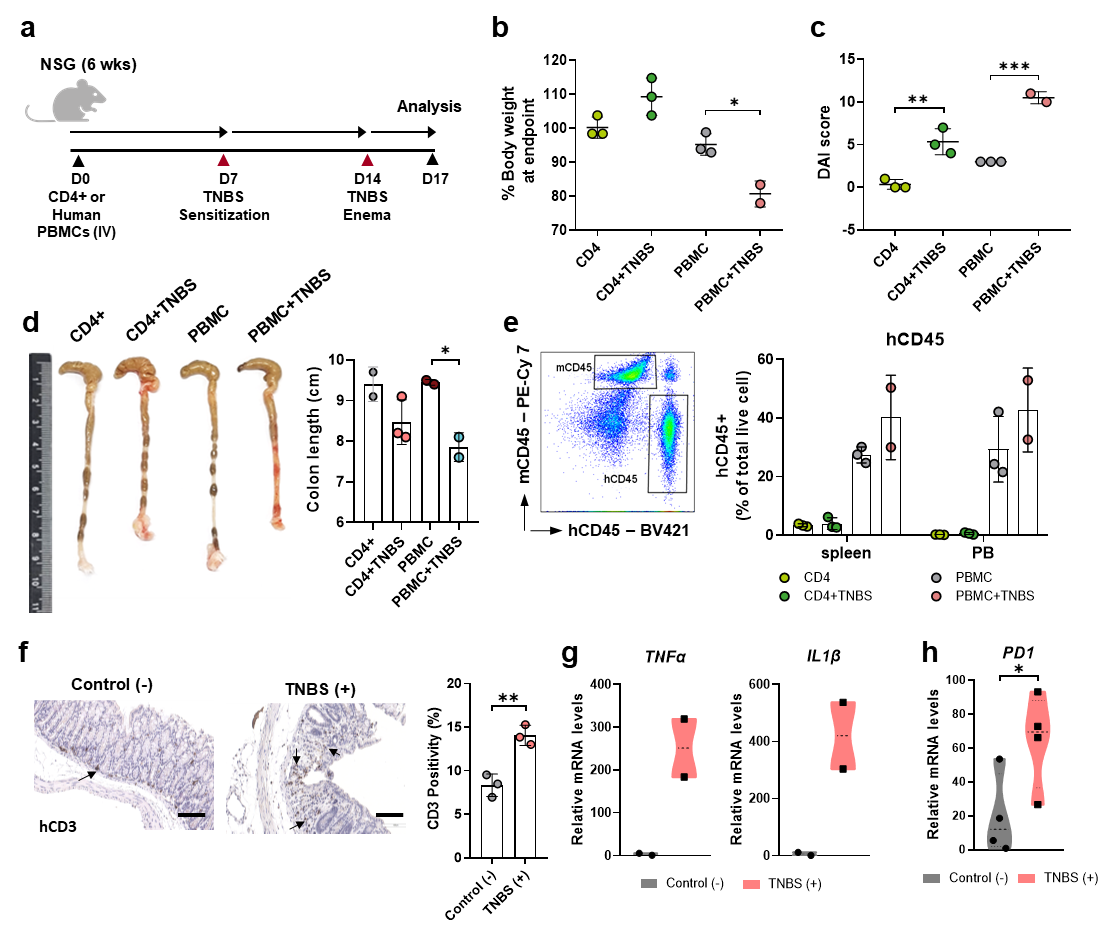


**Supplementary Figure 13.** Pilot establishment of a humanized T cell–mediated colitis model in NSG mice. **a Experimental timeline for TNBS-induced colitis in NSG mice reconstituted with human CD4⁺ T cells or PBMCs. TNBS sensitization and rectal administration were performed as indicated, with endpoint analyses on day 17. b** Body weight changes (%) monitored following TNBS challenge. **c** DAI scores assessed 3 days after TNBS administration. **d** Representative images and quantification of colon length across experimental group. **e** Flow cytometric analysis of human CD45⁺ cell engraftment in the spleen and peripheral blood (PB) of NSG mice. Left, gating strategy; right, percentage of hCD45⁺ cells in each group. **f** Immunohistochemical (IHC) staining for human CD3 in colon sections from PBMC and PBMC + TNBS groups. **g** qRT-PCR analysis of TNF-α and IL-1β mRNA levels in colon tissues. **h** qRT-PCR analysis of PD-1 mRNA expression in colon tissues. Panels (a-h) summarize exploratory pilot experiments (n = 2–3 biological replicates per group) performed to optimize human PBMC engraftment and TNBS induction parameters (dose, timing, feasibility). Data represent mean ± SD. Statistical comparisons were performed using unpaired Student’s t-test or one-way ANOVA with Tukey’s multiple comparisons test. p-values, where indicated, should be interpreted with caution due to limited sample size. Confirmatory experiments with increased replicates (n ≥ 7 per group) are presented in **Fig. 7.**

Figure S14.


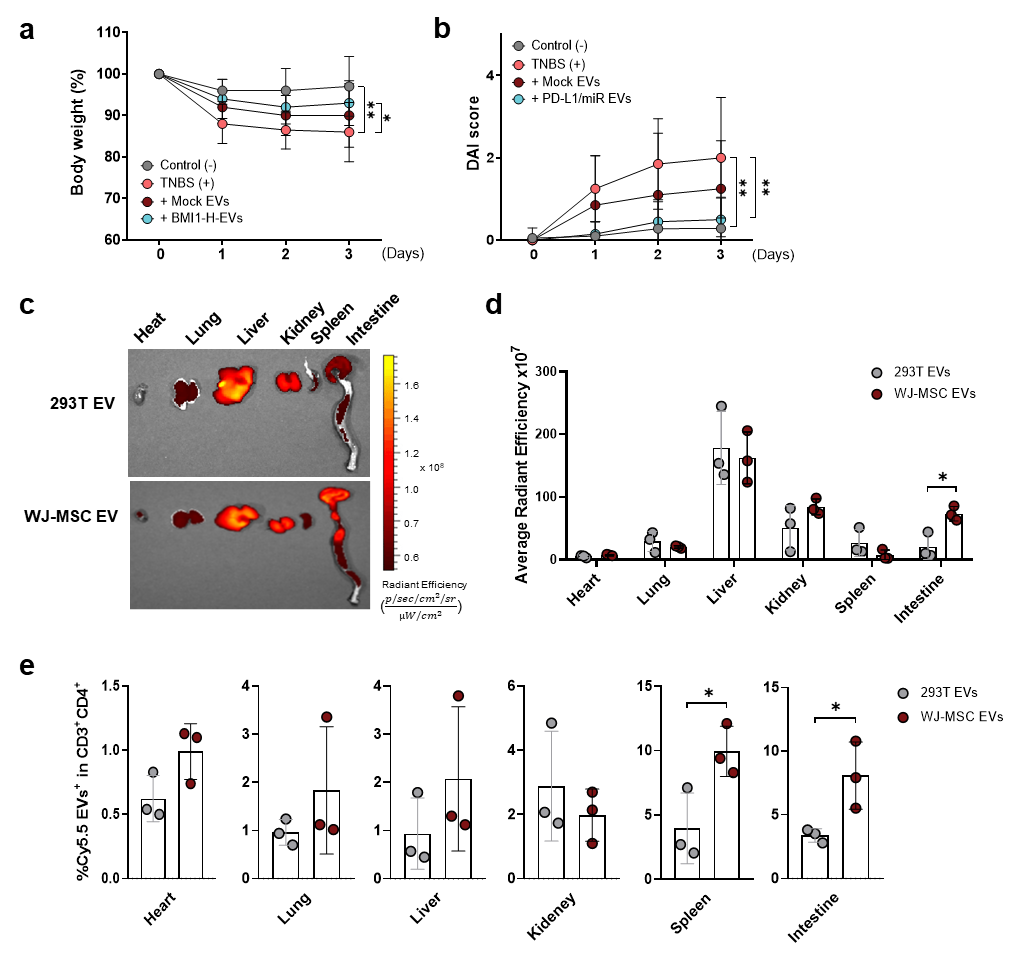


**Supplementary Figure 14.** Biodistribution and uptake of EVs in a TNBS-induced colitis model. **a, b** Time-course analyses of body-weight changes (**a**) and disease activity index (DAI) scores (**b**) in the TNBS-induced colitis model. Body weight and DAI were monitored daily after rectal TNBS administration. Data are presented as mean ± SD from n = 7-15 mice per group (biological replicates). Statistical analysis was performed by two-way repeated-measures ANOVA (treatment × day) followed by Sidak’s multiple comparisons at each time point. **c** Representative IVIS images showing biodistribution of Cy5.5-labeled EVs derived from 293T cells or WJ-MSCs in major organs, including the intestine. **d** Quantification of Cy5.5 fluorescence intensity (average radiant efficiency) in the indicated organs. **e** Flow cytometric analysis of Cy5.5-labeled EV uptake by CD4⁺ T cells isolated from various organs, including the intestine. Data are shown as mean ± SD (n = 3 mice per group, biological replicates). Statistical significance was determined using paired Student’s t-test. * *p* < 0.05 versus 293T EVs, as indicated.**Figure S15.**


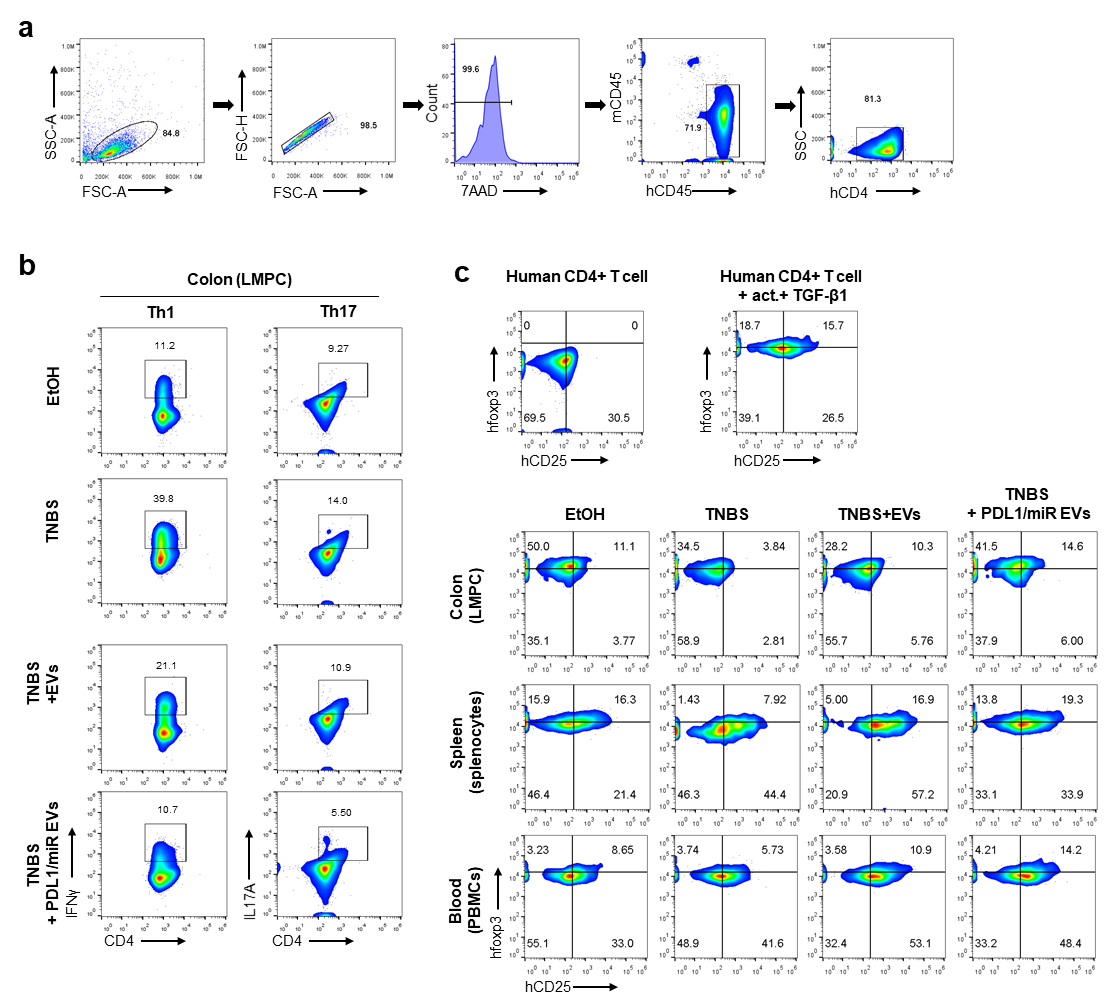


**Supplementary Figure 15.** Regulation of T cell subsets by PD-L1/miR-27a-3p-enriched EVs in humanized TNBS-induced colitis mice. **a** Gating strategy for the identification of activated T cell subsets using human CD4⁺ T cells isolated from the colonic lamina propria (LMPCs). Sequential gating: FSC/SSC → singlets → live (7-AAD^-^) → hCD45⁺/mCD45⁻ → CD3⁺ → CD4⁺. Cells were stimulated with PMA/ionomycin in the presence of brefeldin A and stained for intracellular cytokines to identify CD4⁺CD25⁺FOXP3⁺ Tregs, CD4⁺IFNγ⁺ Th1 cells, and CD4⁺IL-17A⁺ Th17 cells. b Representative flow cytometry plots showing IFNγ⁺ CD4⁺ Th1 and IL-17A⁺ CD4⁺ Th17 cells from the LMPCs of mice treated with EtOH (Control), TNBS, TNBS + EVs, or TNBS + PD-L1/miR EVs. **c** Flow cytometric analysis of CD25⁺FOXP3⁺ Tregs in the colon, spleen, and peripheral blood across treatment groups. Positive control: in vitro TGF-β-induced human Tregs (CD25^high FOXP3⁺); negative control: resting human CD4⁺ T cells (CD25^lowFOXP3⁻). FMO-defined thresholds (CD25-FMO and FOXP3-FMO) were used to establish positivity and applied uniformly across all samples. Data represent mean ± SD from n = 3 mice per group. Flow cytometry was performed in technical triplicates. Quantitative analyses are presented in the main figures.

Figure S16.


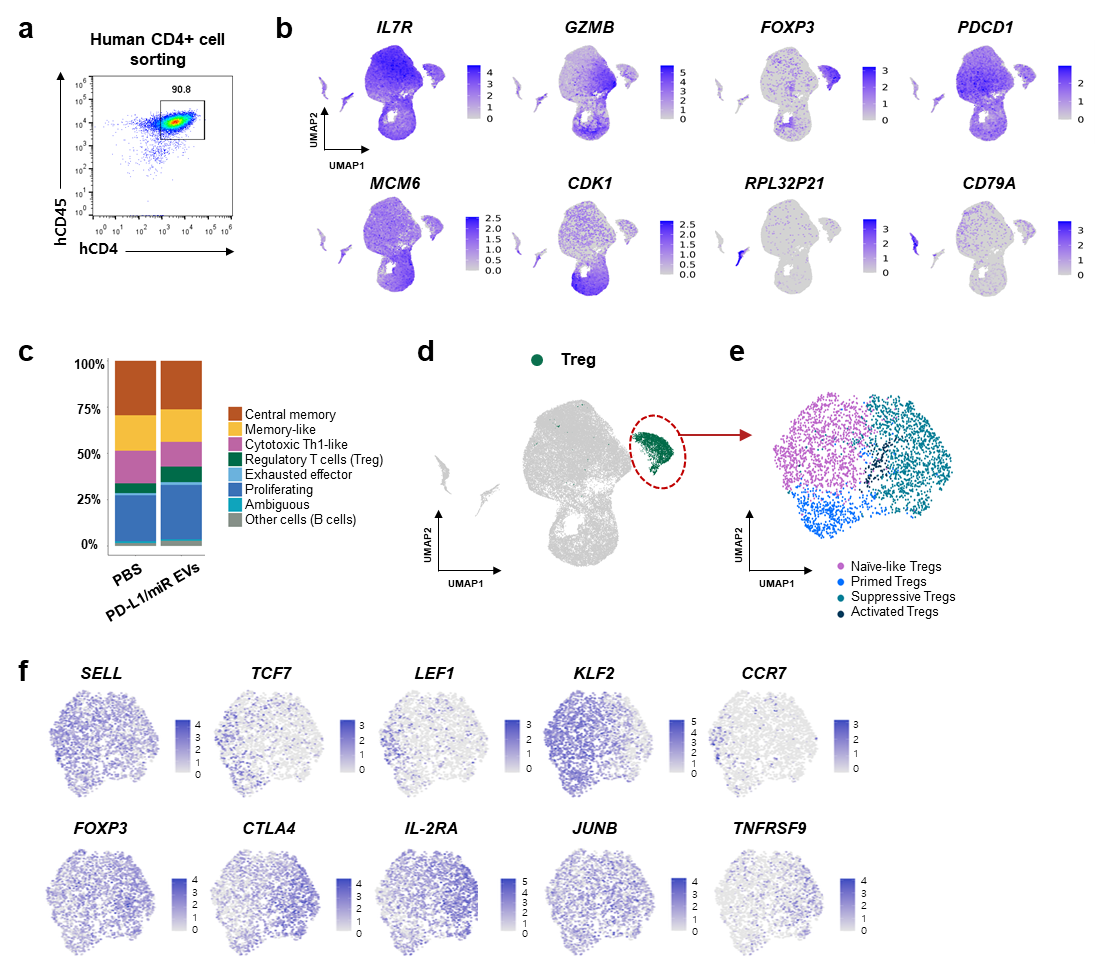


**Supplementary Figure 16.** Single-cell transcriptomic characterization of splenic CD4⁺ T cells following PD-L1/miR-27a-3p EV treatment in humanized colitis mice. **a** Flow cytometry plot confirming >90% purity of isolated human CD4⁺ T cells used for single-cell RNA-seq. **b** UMAP feature plots illustrating the expression of representative markers for cell type annotation: IL7R (naïve/memory), GZMB (cytotoxic), FOXP3 (regulatory T cell), PDCD1 (exhausted), and proliferation-associated genes (MCM6, CDK1, RPL32P21). **c** Bar plot showing the relative proportions of identified CD4⁺ T cell clusters in PBS- and PD-L1/miR-27a-3p EV-treated groups. **d** UMAP plot highlighting the regulatory T cell cluster extracted for subclustering analysis. **e** UMAP visualization of four Treg subsets identified by subclustering: naïve-like, primed, suppressive, and activated Tregs. **f** Feature plots showing the expression levels of representative genes across Treg subsets. Single-cell RNA-seq performed on pooled splenic CD4⁺ T cells from spleen of n = 3 human donor per group (biological replicates). Each group was analyzed as one scRNA-seq library. Cluster proportions compared descriptively; no multiple-testing correction applied.

**Supplementary Tables**

**Table S1. Clinical characteristics of IBD patients**

| **Patient ID** | **Initial Diagnosis** | **Sex** | **Age** | **Disease duration (year)** | **Disease location** | **Disease behavior** | **Disease activity (Mayo/CDAI)** | **Treatment at sampling** | **Clinical Status at Sampling** | **CRP / ESR** |  |
| --- | --- | --- | --- | --- | --- | --- | --- | --- | --- | --- | --- |
| Patient 1 | CD | male | 22 | 9 | L2 | B2 | 50 | IFX | Remission | h-crp 0.17 ESR/ 10 |  |
| Patient 2 | UC | male | 23 | 1 | Pancolitis |  | 7 | filgotinib | active | h-crp 0.02 ESR/ 4 |  |
| Patient 3 | CD | male | 21 | 9 | L2 | B1 | 0 | IFX | Remission | h-crp 0.05 ESR/ 6 |  |
| **Diagnosis**: Ulcerative colitis (UC) and Crohn's disease (CD); **Disease location(L)**: L1 terminla ileum, L2 colon, L3 ileocolon, L4 upper gastrointestinal tract; **Disease behavior(B)**: B1 non stricturing nonpenetrating, B2 stricturing, B3 penetrating | | | | | | | | | | | |

**Table S2. The number of generated cells**

|  | **PBS** | | | **PD-L1/miR EV** | | |
| --- | --- | --- | --- | --- | --- | --- |
| **Sample** | S1 | S2 | Total | S1 | S2 | Total |
| **Cell numbers after QC** | 12,081 | 12,220 | 24,301 | 11,927 | 12,227 | 24,154 |
| **Filtering rate** | 8.53% | 8.22% | 8.37% | 9.35% | 9.35% | 9.35% |
| **Filtered cell numbers** | 11,050 | 11,216 | 22,266 | 10,812 | 11,084 | 21,896 |

**Table S3. Differential gene expression of CD4^+^ cells.**

Data provided in Excel file Supplementary Table S3.

**Table S4. Cell numbers of each cell type in CD4 T cell population.**

| **Condition** | **PBS** | **PBS** | **PBS** | **PBS** | **PBS** | **PBS** | **PBS** | **PBS** | **PBS** |
| --- | --- | --- | --- | --- | --- | --- | --- | --- | --- |
| **Cell type** | Central memory CD4 T cells | Memory-like CD4 T cells | Cytotoxic Th1-like CD4 T cells | Regulatory T cells (Tregs) | Exhausted effector CD4 T cells | Proliferating CD4 T cells | Ambiguous CD4 T cells | B cells (others) | Total |
| **Total** | 6,544 | 4,272 | 3,921 | 1,173 | 264 | 5,522 | 225 | 345 | 22,266 |
|  |  |  |  |  |  |  |  |  |  |
| **Condition** | **PD_L1/miR EV** | **PD_L1/miR EV** | **PD_L1/miR EV** | **PD_L1/miR EV** | **PD_L1/miR EV** | **PD_L1/miR EV** | **PD_L1/miR EV** | **PD_L1/miR EV** | **PD_L1/miR EV** |
| **Cell type** | Central memory CD4 T cells | Memory-like CD4 T cells | Cytotoxic Th1-like CD4 T cells | Regulatory T cells (Tregs) | Exhausted effector CD4 T cells | Proliferating CD4 T cells | Ambiguous CD4 T cells | B cells (others) | Total |
| **Total** | 5,740 | 3,848 | 2,923 | 1,838 | 332 | 6,432 | 175 | 608 | 21,896 |

**Table S5. Differential gene expression of Treg subsets.**

Data provided in Excel file Table S5.

**Table S6. Differentially expressed genes for PD-L1/miR EV vs PBS in the Treg population**

Data provided in Excel file Table S6.

**Table S7. Disease Activity Index (DAI) rubric for DSS colitis (0–12)**

| **Category** | **Score 0** | **Score 1** | **Score 2** | **Score 3** | **Score 4** |
| --- | --- | --- | --- | --- | --- |
| Weight loss (% vs. day 0) | <1% | 1–5% | >5–10% | >10–20% | >20% |
| Stool consistency | Normal, formed pellets | Mild soft | Loose stools | — | Diarrhea (liquid, no form) |
| Fecal blood | Negative | slightly bedding contamination | Occult blood positive | slightly bleeding | Gross rectal bleeding/hematochezia |

**Table S8. Clinical score rubric for TNBS colitis (0–12)**

| **Category** | **Score 0** | **Score 1** | **Score 2** | **Score 3** | **Score 4** |
| --- | --- | --- | --- | --- | --- |
| Weight change (% from day 0, post-TNBS enema) | <1% | 1–5% | >5–10% | >10–20% | >20% |
| Activity / posture | Active, normal grooming | Slightly reduced activity | Lethargic; mild hunching | Marked hunching; reluctant to move | Immobile or moribund |
| Stool / anal findings | Normal pellets; clean perianal area | Soft stool; minimal soiling | Loose stool; mild perianal soiling | Watery diarrhea; clear soiling | Profuse diarrhea; severe soiling/prolapse |

**References**

1 Kang, J. Y. *et al.* Xeno-Free Condition Enhances Therapeutic Functions of Human Wharton's Jelly-Derived Mesenchymal Stem Cells against Experimental Colitis by Upregulated Indoleamine 2,3-Dioxygenase Activity. *J Clin Med* **9** (2020).

2 Joo, H. *et al.* Extracellular Vesicles from Thapsigargin-Treated Mesenchymal Stem Cells Ameliorated Experimental Colitis via Enhanced Immunomodulatory Properties. *Biomedicines* **9** (2021).

3 Lun, A. T. L. *et al.* EmptyDrops: distinguishing cells from empty droplets in droplet-based single-cell RNA sequencing data. *Genome Biol* **20**, 63 (2019).

4 Lun, A. T., McCarthy, D. J. & Marioni, J. C. A step-by-step workflow for low-level analysis of single-cell RNA-seq data with Bioconductor. *F1000Res* **5**, 2122 (2016).

5 Stuart, T. *et al.* Comprehensive Integration of Single-Cell Data. *Cell* **177**, 1888-1902.e1821 (2019).

6 Korsunsky, I. *et al.* Fast, sensitive and accurate integration of single-cell data with Harmony. *Nat Methods* **16**, 1289-1296 (2019).

7 Heaton, H. *et al.* Souporcell: robust clustering of single-cell RNA-seq data by genotype without reference genotypes. *Nat Methods* **17**, 615-620 (2020).

**Uncropped western blots**


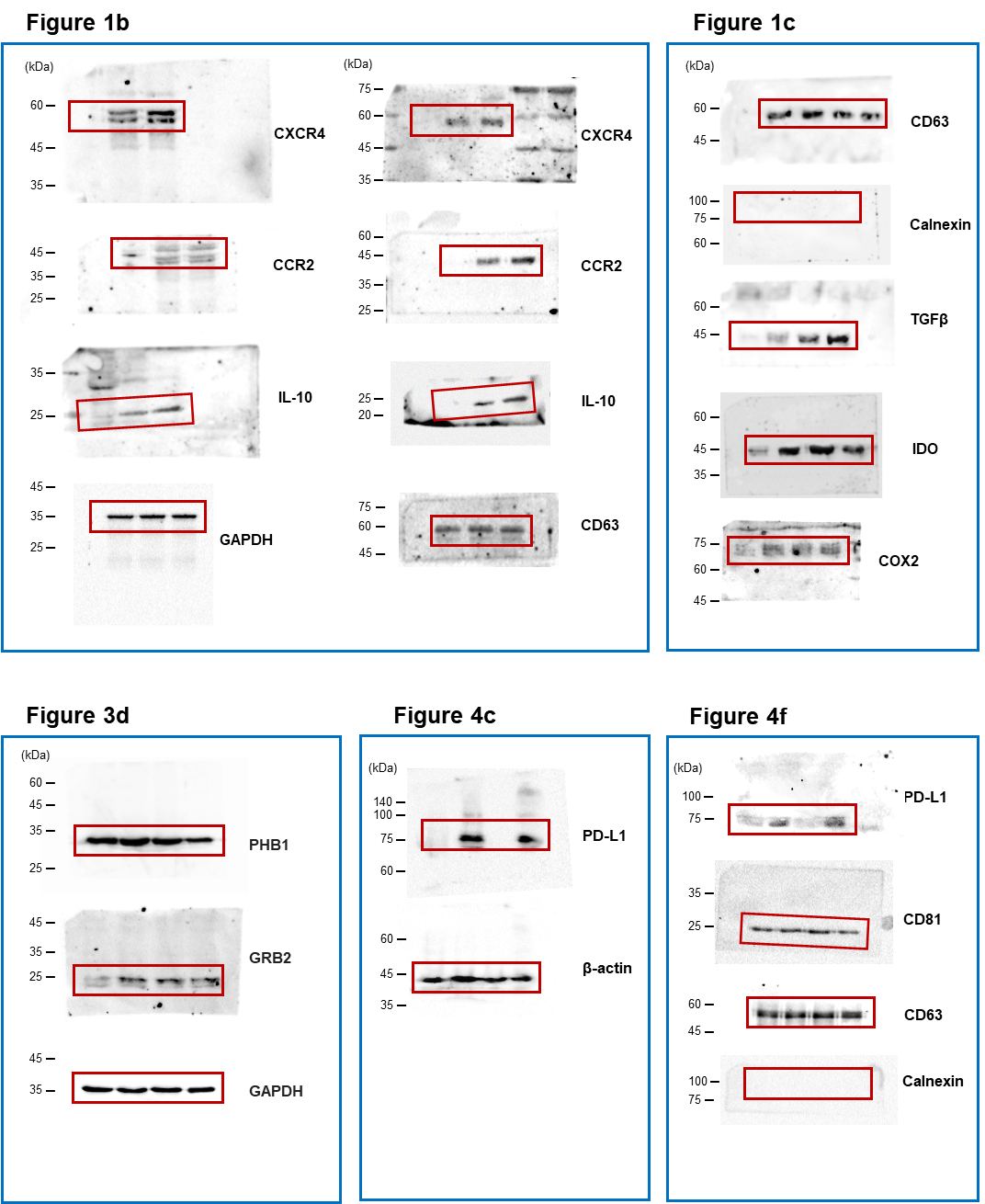


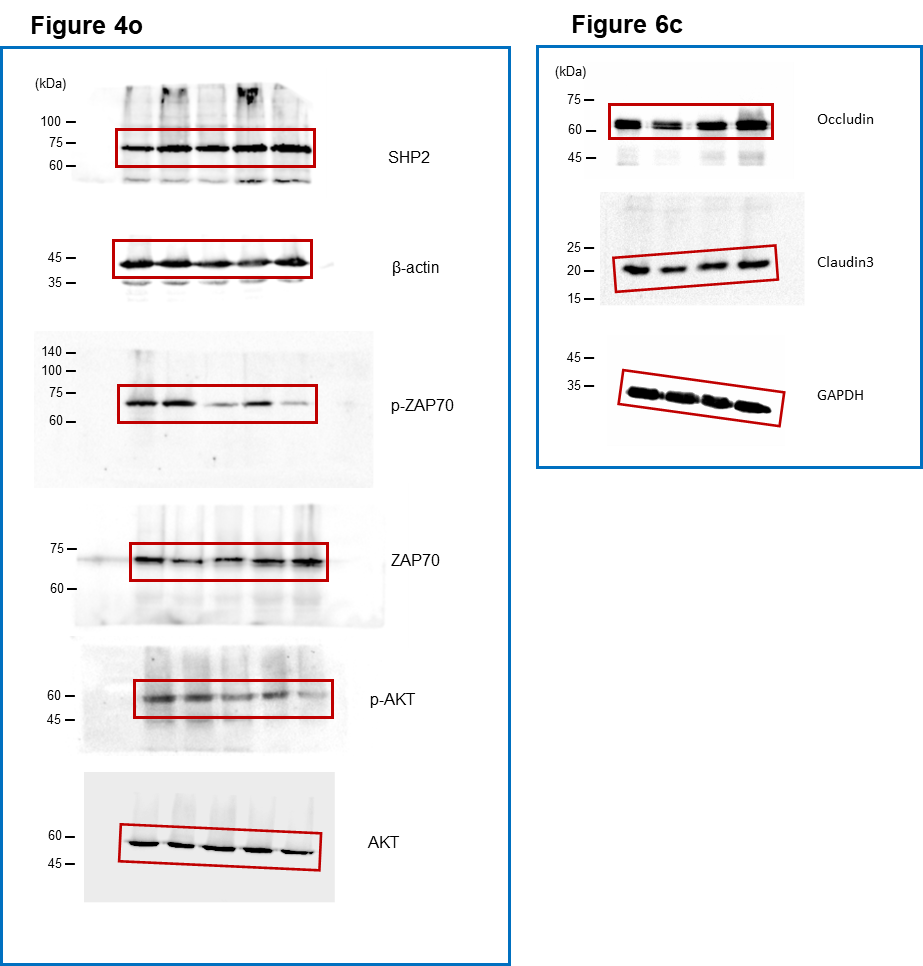


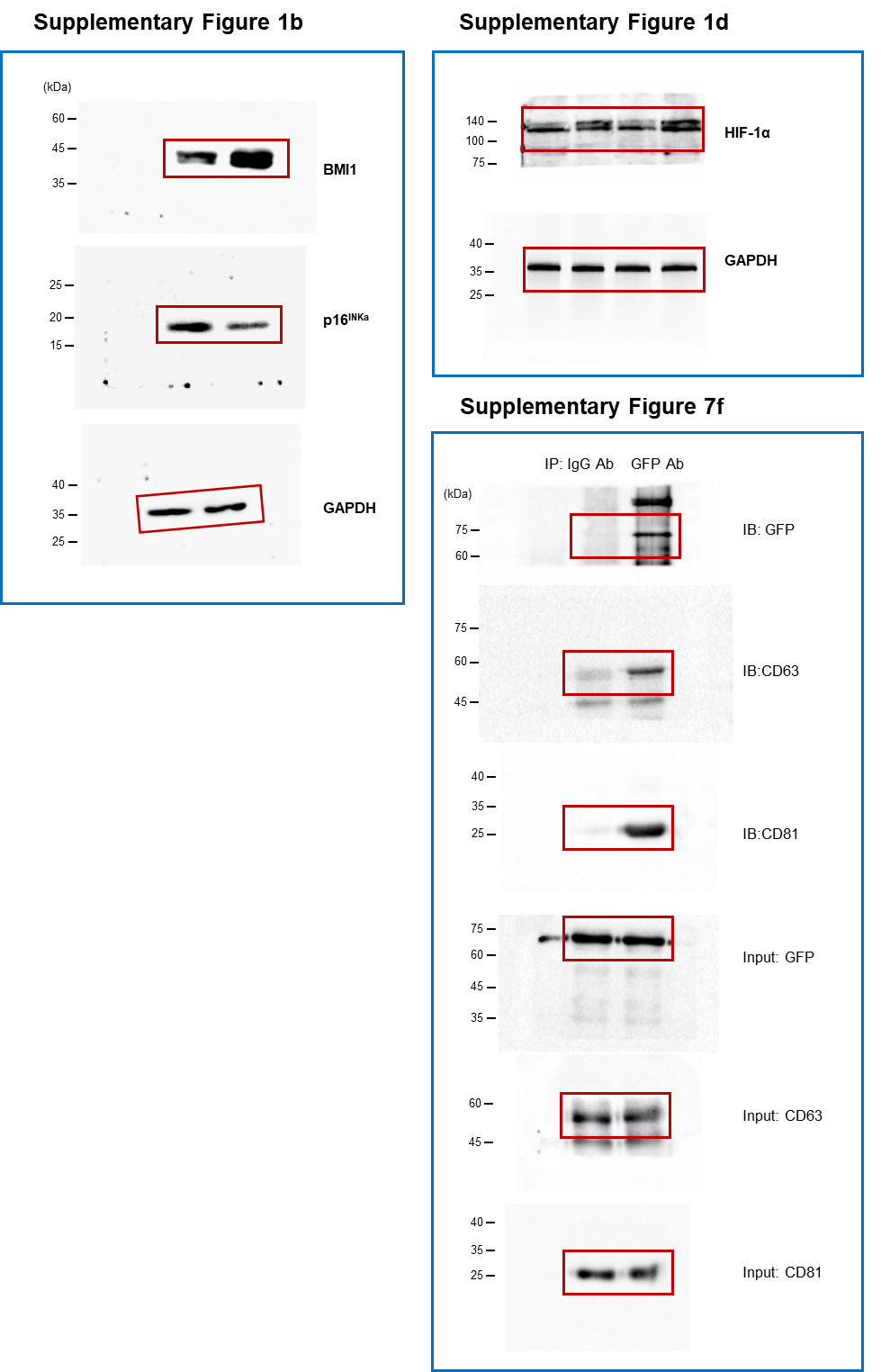

Supplement: Supplementary file 1 — Revised Supplementary Materials [file 41392_2025_2516_MOESM1_ESM.docx]
